# Time-Series Transcriptomics Reveals Temporal Expression Patterns in Soybean (*Glycine max*) Roots During Symbiosis with *Bradyrhizobium ottawaense* HK601

**DOI:** 10.3390/plants15213420

**Published:** 2026-11-03

**Authors:** Jianan Zhu, Yingqi Sun, Liqiang Meng, Xiangxiang Wang, Xinkai Ma, Chao Jiang

**Affiliations:** Institute of Microbiology, Heilongjiang Academy of Sciences, 68 Zhaolin Street, Harbin 150010, China; 18846921667@163.com (J.Z.); sunyq200112@163.com (Y.S.); mengliqiang83420@163.com (L.M.); 18946115901@163.com (X.W.); m1963710204@163.com (X.M.)

**Keywords:** soybean (*Glycine max*), symbiotic nitrogen fixation, temporal transcriptomics, isoflavonoid biosynthesis, plant MAPK signaling pathway

## Abstract

Rhizobium–soybean symbiosis plays a critical role in sustainable agriculture. We isolated the slow-growing strain HK601 from the soybean cultivar ‘Heike 60’ in cold-region Heilongjiang, China, and identified it as *Bradyrhizobium ottawaense* by four-gene phylogeny. In pot trials, HK601 inoculation significantly promoted plant growth, effective nodule number, and total nitrogen content. HK601 also formed effective nodules on Williams 82 and 11 Heilongjiang cultivars, showing broad-spectrum nodulation. Time-series transcriptomes of symbiotic soybean roots showed 14 dpi was a key turning point in transcriptional reprogramming. Before 14 dpi, signal transduction and nodule structural development DEGs were active. After 14 dpi, DEGs related to ribosome assembly and translation declined, whereas DEGs involved in isoflavonoid biosynthesis, MAPK signaling, and defense-related pathways were transcriptionally up-regulated. This complementarity may reflect a shift from basal metabolism to symbiotic functions. The temporally cascaded activation of isoflavonoid and MAPK pathways during the mid-to-late stages may reflect a potential dual role in symbiotic signaling and defense, thereby contributing to symbiotic homeostasis. Sharp up-regulation of symbiotic marker genes (leghemoglobin and nodulins) after 14 dpi provided transcriptional evidence for the establishment of symbiotic nitrogen fixation. This study provides a promising candidate strain for cold-region soybean production and a new transcriptomic perspective on temporal host–rhizobia regulation.

## 1. Introduction

*Glycine max* (soybean) is a major economic crop worldwide, valued for its high protein and oil content, and plays a key role in both human nutrition and animal feed [1]. Soybean production relies heavily on nitrogen availability, but its symbiotic nitrogen fixation with rhizobia can effectively alleviate this demand. This system supplies more than 50% of the nitrogen required by soybean plants, and as a major contributor to global biological nitrogen fixation, rhizobia account for over 65% of the total fixed nitrogen [2]. However, the symbiotic relationship between rhizobia and soybean is highly host-specific, meaning that a given rhizobial strain can only nodulate one or a limited number of soybean genotypes. Introduced rhizobial inoculants often perform poorly due to regional variation in soybean cultivars, diverse native rhizobia, and their competitive interactions [3]. To address this bottleneck, we focused on major soybean cultivars in Heilongjiang Province, the largest soybean-producing region in China, and screened for rhizobial strains with high compatibility and strong adaptability.

Among rhizobial resources, *Bradyrhizobium ottawaense* has recently gained attention as a novel symbiotic nitrogen-fixing bacterium associated with soybean. It was first isolated and characterized from soybean nodules in the Ottawa region of Canada. It forms effective nodules on a range of legumes, including soybean (*G. max*), wild soybean (*G. soja*), and *Macroptilium atropurpureum* [4]. Notably, it also exhibits high nitrous oxide (N_2_O) reductase activity, making it a promising strain for both soybean nitrogen supply and mitigation of agricultural greenhouse gas emissions [5]. *B. ottawaense* shows a regionally restricted distribution, having been recovered from only a limited number of sampling sites in Japan [5]. In China, however, *B. ottawaense* has so far been reported only from peanut (*Arachis hypogaea* L.) nodules in Henan Province [6]. Its occurrence and symbiotic performance in cold-region soybean areas, such as Heilongjiang, remain unexamined. Therefore, this study carried out local isolation and systematic evaluation of this species, aiming to obtain indigenous *B. ottawaense* and assess its symbiotic nodulation performance.

The establishment of soybean–rhizobia symbiosis involves several sequential stages, including root hair recognition, infection thread formation, cortical cell division, nodule primordium development, and nodule maturation [7,8]. Flavonoids are secreted by soybean roots and perceived by rhizobia. This induces the expression of rhizobial nodulation genes (nod genes) and initiates symbiotic signal exchange [9,10]. Nod factors released by rhizobia are directly recognized by the nod factor receptors *NFR1* and *NFR5* on the soybean root plasma membrane [11,12]. This recognition recruits the core co-receptor SYMRK, which transduces the extracellular signal across the membrane and activates downstream phosphorylation cascades [13,14,15]. This cascade amplifies the symbiotic signal through the MAPK pathway and relays it to the nucleus, where it initiates transcription of nodulation-related genes and promotes nodule development and maturation [16]. In late nodule development, the expression of nodulin and leghemoglobin genes marks the transition to maturity [17,18]. Nodulins contribute to nodule structural establishment [19], while leghemoglobin maintains a microaerobic environment to support nitrogenase activity [20].

With the rapid development of high-throughput sequencing, transcriptome analysis has become a key approach for dissecting the molecular mechanisms of plant–microbe interactions and is now widely applied in symbiotic nitrogen fixation research. Many studies have used this approach to identify differentially expressed genes and enriched pathways in soybean after rhizobial inoculation. These findings have provided a molecular basis for understanding symbiotic nitrogen fixation [21,22]. However, most studies have relied on conventional transcriptome sequencing at a single or a few time points. Such static comparisons do not effectively capture the dynamic changes in host gene expression during symbiosis. Moreover, most transcriptome studies have focused on early infection events within hours to days after inoculation. Only DelPercio et al. [23] performed a time-series analysis covering nodule development through senescence in soybean inoculated with *B. diazoefficiens* USDA110. That study provided useful insights into the transcriptional regulation of nodule development. However, that study focused on nodule-localized dynamics, leaving the temporal transcriptomic changes in whole roots largely unexplored. In addition, one study compared the soybean transcriptome at 3 dpi after inoculation with *B. ottawaense* versus *B. diazoefficiens* [24]. This analysis was restricted to a single early time point. The full temporal dynamics of the soybean root transcriptome after *B. ottawaense* inoculation have not been systematically described.

To address this gap, this study isolated rhizobia from nodules of ‘Heike 60’, a major soybean cultivar in Heilongjiang Province, and identified *B. ottawaense* by multilocus phylogenetic analysis. The symbiotic nodulation and nitrogen fixation characteristics, as well as host broad-spectrum compatibility, were systematically evaluated to provide a germplasm basis for screening cold-adapted strains. On this basis, transcriptome sequencing was performed on soybean roots at 0, 2, 7, 14, 21, and 28 days post-inoculation (dpi) with this strain. This approach aims to reveal the temporal transcriptomic patterns during the establishment of symbiotic nitrogen fixation and to provide a new transcriptomic perspective on the time-course regulation of host–rhizobia interactions.

## 2. Results

### 2.1. Morphological and Phylogeny Characteristics

**Figure 1:**
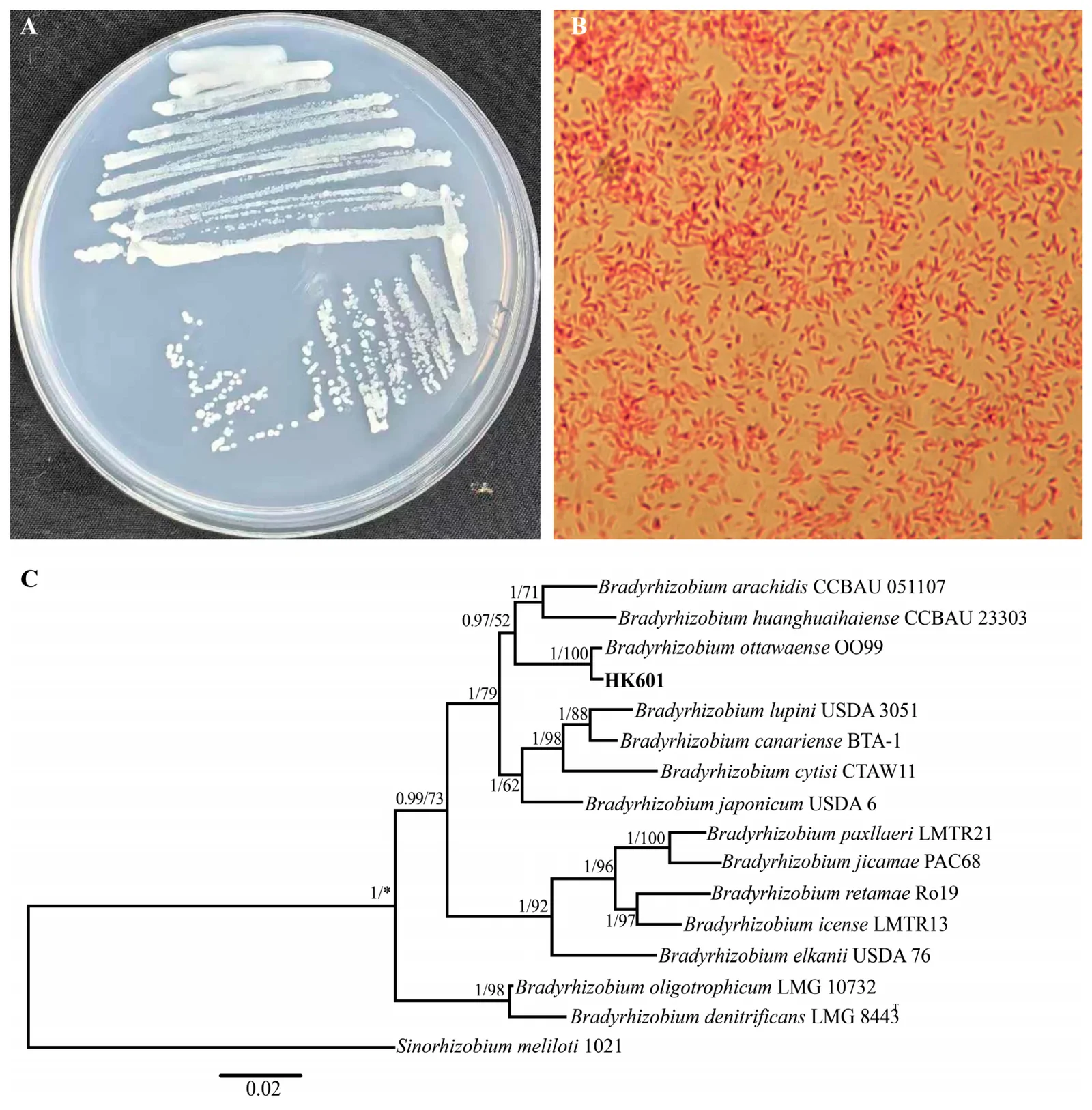
Morphology and phylogenetic analysis of *B. ottawaense* HK601. (**A**) Colony morphology of strain HK601 grown on YMA medium at 28 °C for 5–7 days. (**B**) Gram staining of strain HK601 observed under a light microscope at 100× magnification. (**C**) Phylogenetic tree based on the concatenated sequences of four genes (*16S rDNA*, *glnII*, *atpD*, and *recA*) constructed using maximum likelihood and Bayesian methods.

The strain HK601 was isolated as a single colony from root nodules of the soybean cultivar ‘Heike 60’ (HK60). After incubation on YMA medium at 28 °C for 5 d, the colonies appeared milky white and circular, with smooth and mucoid surfaces (Figure 1A). Gram staining resulted in red cells, a typical feature of Gram-negative bacteria (Figure 1B). To determine the phylogenetic position of strain HK601, the *16S rRNA* gene, *glnII*, *atpD*, and *recA* were sequenced. A concatenated phylogenetic tree based on the four genes was constructed using both maximum likelihood and Bayesian methods. The two trees showed identical topology, and strain HK601 was consistently grouped with *B. ottawaense* with 100% bootstrap support. Based on the morphological characteristics and phylogenetic analysis, the strain HK601 was identified as *B. ottawaense* (Figure 1C).

### 2.2. Potting Inoculation Effect and Host Range of B. ottawaense HK601

**Figure 2:**
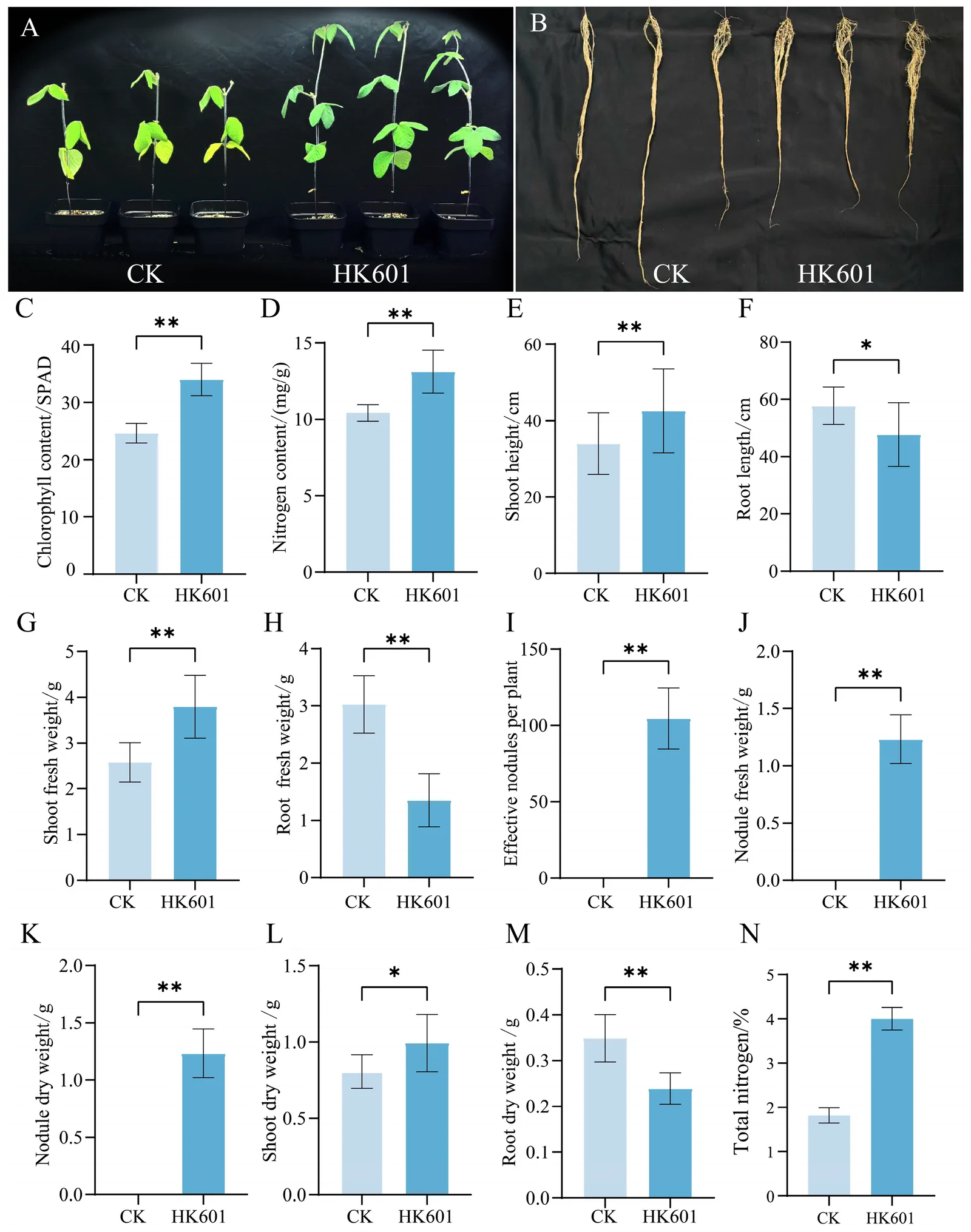
Symbiotic compatibility test between *B. ottawaense* HK601 and its homologous host soybean cultivar ‘Heike 60’. (**A**) Soybean plants of cultivar ‘Heike 60’ at 28 days after inoculation with HK601. (**B**) Soybean roots at 28 days after inoculation with HK601. (**C**) Leaf chlorophyll content (SPAD value). (**D**) Leaf nitrogen content. (**E**) Shoot height. (**F**) Root length. (**G**) Shoot fresh weight. (**H**) Root fresh weight (excluding nodules). (**I**) Number of effective nodules per plant. (**J**) Fresh weight of effective nodules. (**K**) Dry weight of effective nodules. (**L**) Shoot dry weight. (**M**) Root dry weight. (**N**) Total nitrogen content of soybean plants. Data were presented as means ± SD (*n* = 15, one plant per pot). * and ** indicate significant differences at *p* < 0.05 and *p* < 0.01 levels, respectively.

Inoculation of the soybean cultivar ‘Heike 60’ with *B. ottawaense* HK601 significantly increased chlorophyll content (Figure 2C), leaf nitrogen content (Figure 2D), shoot height (Figure 2E), shoot fresh weight (Figure 2G), and shoot dry weight (Figure 2L) by 33.70%, 25.14%, 43.00%, 49.73%, and 23.95%, respectively, compared to the non-inoculated control at 28 dpi (Figure 2A,B). Inoculation with *B. ottawaense* HK601 also significantly promoted nodulation in ‘HK60’. The number of effective nodules reached 101.00 ± 17.65 per plant (Figure 2I). The fresh weight and dry weight of effective nodules were 0.53 ± 0.12 g (Figure 2J) and 0.13 ± 0.16 g (Figure 2K), respectively. Inoculation with HK601 also effectively increased the total nitrogen content in soybean plants by 121.11% (Figure 2N) compared to the uninoculated control, further validating its nitrogen-fixing potential as an excellent rhizobium strain. In addition, *B. ottawaense* HK601 was inoculated onto the soybean cultivar Williams 82 and 11 other soybean cultivars from different accumulated temperature zones in Heilongjiang Province, China (Figure S1). Effective nodules (pink nodules) were observed to varying degrees across all tested cultivars (Figure S2G), indicating that HK601 has a broad-spectrum nodulation characteristic. Moreover, inoculation with *B. ottawaense* HK601 increased chlorophyll content (Figure S2A), shoot fresh weight (Figure S2E) and nodule fresh weight (Figure S2H) to varying degrees across the tested cultivars.

### 2.3. Quality Assessment of RNA-Seq Data and Sample Cluster Analysis

**Figure 3:**
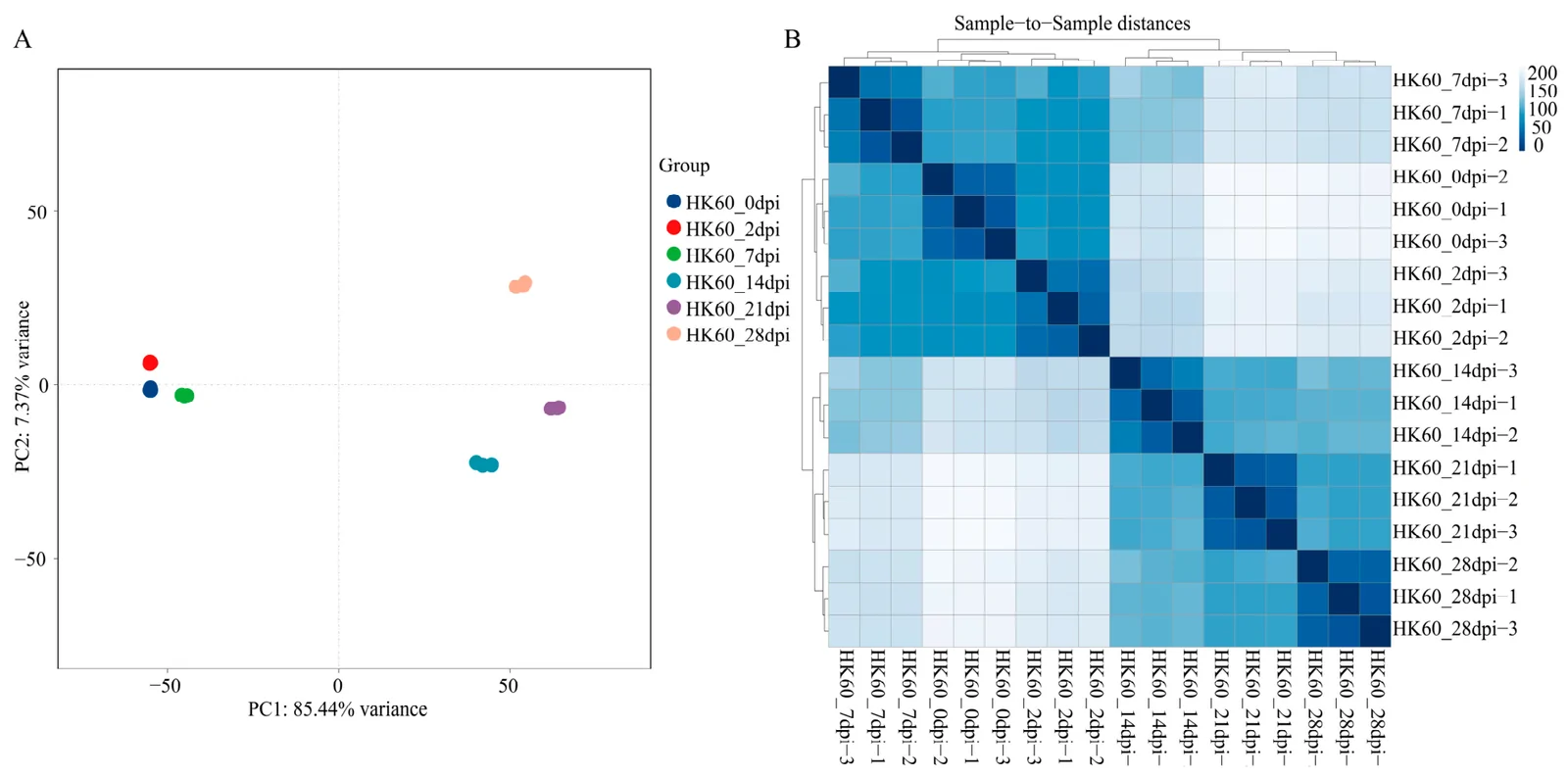
Quality assessment of transcriptome samples. (**A**) Principal component analysis (PCA) of all samples based on gene expression profiles. (**B**) Sample-to-sample distance heatmap based on Euclidean distance.

We performed RNA-seq on 18 root samples of soybean inoculated with *B. ottawaense* HK601 at six time points (0, 2, 7, 14, 21, and 28 dpi), with three biological replicates per time point. The raw data output for each sample ranged from 6.99 to 7.14 Gb. After quality filtering, more than 23 million clean reads were obtained per sample. The Q30 base quality ratio ranged from 98.10% to 98.60%, and the GC content ranged from 39.56% to 43.93%. More than 97% of the clean reads mapped to the reference genome, with 93.98–96.05% of them being unique mappings, indicating good sequencing data quality (Table S1). PCA results showed that PC1 and PC2 explained 85.44% and 7.37% of the total expression variance, respectively. The three biological replicates at each time point clustered closely together, and samples from different time points were clearly separated along PC1 (Figure 3A), indicating reliable sample grouping and high reproducibility. The sample-to-sample distance heatmap further showed that the 18 samples were separated into two distinct clusters (Figure 3B). The HK60-0 dpi, HK60-2 dpi, and HK60-7 dpi form one group, while the HK60-14 dpi, HK60-21 dpi, and HK60-28 dpi form another group. The clustering distance between the two branches was greater than that within each branch. Further analysis revealed that transcriptional changes in soybean roots were relatively weak from 0 to 7 dpi after inoculation, whereas a significant transcriptional reprogramming was initiated from 14 dpi onward and progressed in a continuous and time-dependent manner.

### 2.4. Temporal Dynamics of DEGs in Soybean Roots After B. ottawaense HK601 Inoculation

**Figure 4:**
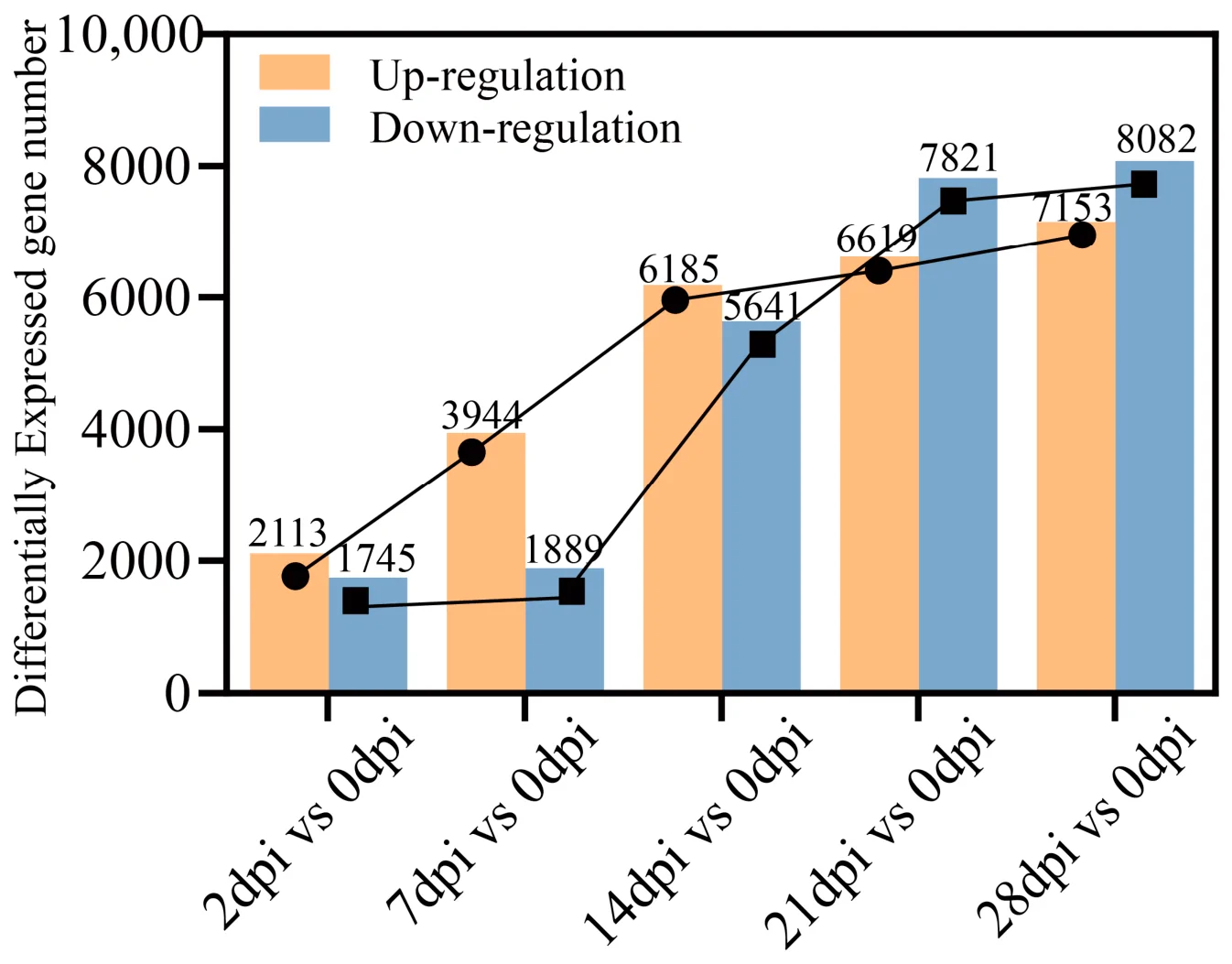
Temporal dynamics of differentially expressed genes in soybean roots after inoculation with HK601.

RNA-seq was performed on soybean roots at different time points after inoculation with HK601. Differentially expressed genes (DEGs) were identified using 0 dpi as the baseline, with a threshold of *q*-value < 0.05 and |log_2_FC| > 1. The total number of DEGs increased from 3858 at 2 dpi to 15,235 at 28 dpi, showing a continuous upward trend as symbiosis progressed (Figure 4). Up-regulated genes increased from 2113 at 2 dpi to 7153 at 28 dpi. Down-regulated genes numbered 1745 and 1889 at 2 and 7 dpi, respectively, then rose sharply to 5641 at 14 dpi and further increased to 7821 and 8082 at 21 and 28 dpi. Notably, at 7 dpi, the number of up-regulated genes was 2.09 times that of down-regulated genes, indicating a predominance of gene activation at this early stage. However, after 21 dpi, the number of down-regulated genes exceeded that of up-regulated genes (Figure 4). Together, these results show that *B. ottawaense* HK601 inoculation triggered three temporal phases in the soybean root transcriptome: an early activation phase (2–7 dpi), a mid-phase suppression onset at 14 dpi, and a late-phase regulatory refinement from 21 to 28 dpi, in line with the PCA and clustering patterns (Figure 3).

### 2.5. Temporal Expression Trend Analysis of Differentially Expressed Genes

**Figure 5:**
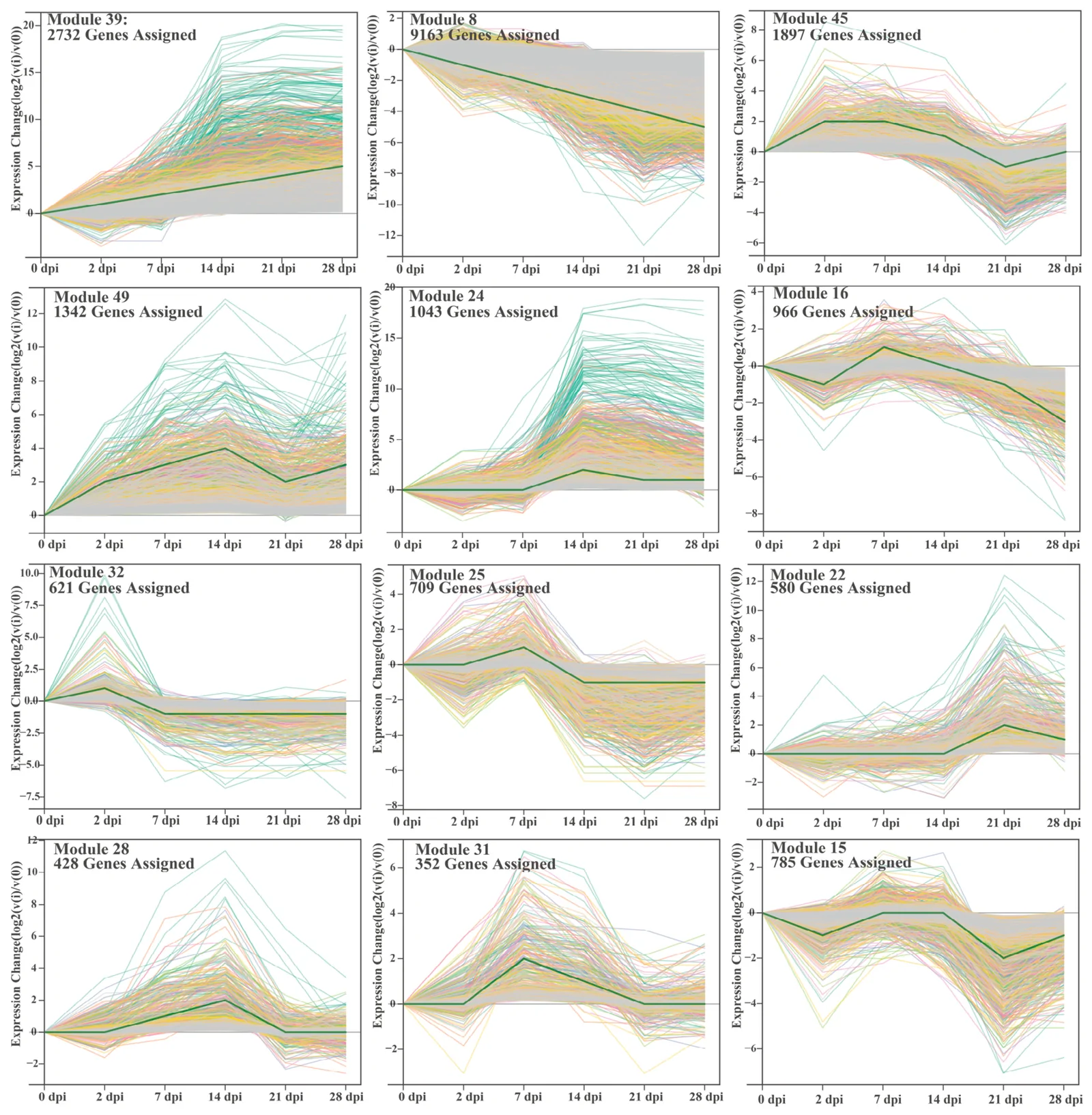
Trend clustering analysis of differentially expressed genes.

To identify temporal expression patterns, we performed trend analysis on DEGs from soybean roots at 0, 2, 7, 14, 21, and 28 dpi. Gene expression levels were normalized to the 0 dpi baseline, and a total of 12 significant expression model profiles were identified (Figure 5). Among these, two modules showed continuous expression changes. Among the 12 modules, Module 39 (2732 DEGs) showed sustained up-regulation that intensified after 14 dpi and peaked at 28 dpi (Figure S3). Module 8 (9163 DEGs) showed a different pattern. It peaked at 2 dpi, then declined steadily, falling below the overall mean after 14 dpi and reaching its lowest point at 28 dpi (Figure S3). In the early-peaking group, Module 32 (621 DEGs) reached its highest expression at 2 dpi. Modules 16, 25, and 31 each exhibited peak expression at 7 dpi, followed by a plateau or a gradual decline thereafter. Among the mid-peaking modules, Modules 49, 24, and 28 all reached their peak expression at 14 dpi, indicating that 14 dpi serves as a critical node for transcriptional regulation of the genes within these three modules. Module 22 contained 580 genes, which showed low expression levels from 0 to 14 dpi. Expression then gradually increased after 14 dpi, peaked at 21 dpi, and declined thereafter. In addition, Modules 45 and 15 contained 1897 and 785 DEGs, respectively. Both modules exhibited fluctuating expression patterns without a distinct peak.

### 2.6. GO and KEGG Enrichment Analysis of Genes in the Continuously Up-Regulated Module 39

**Figure 6:**
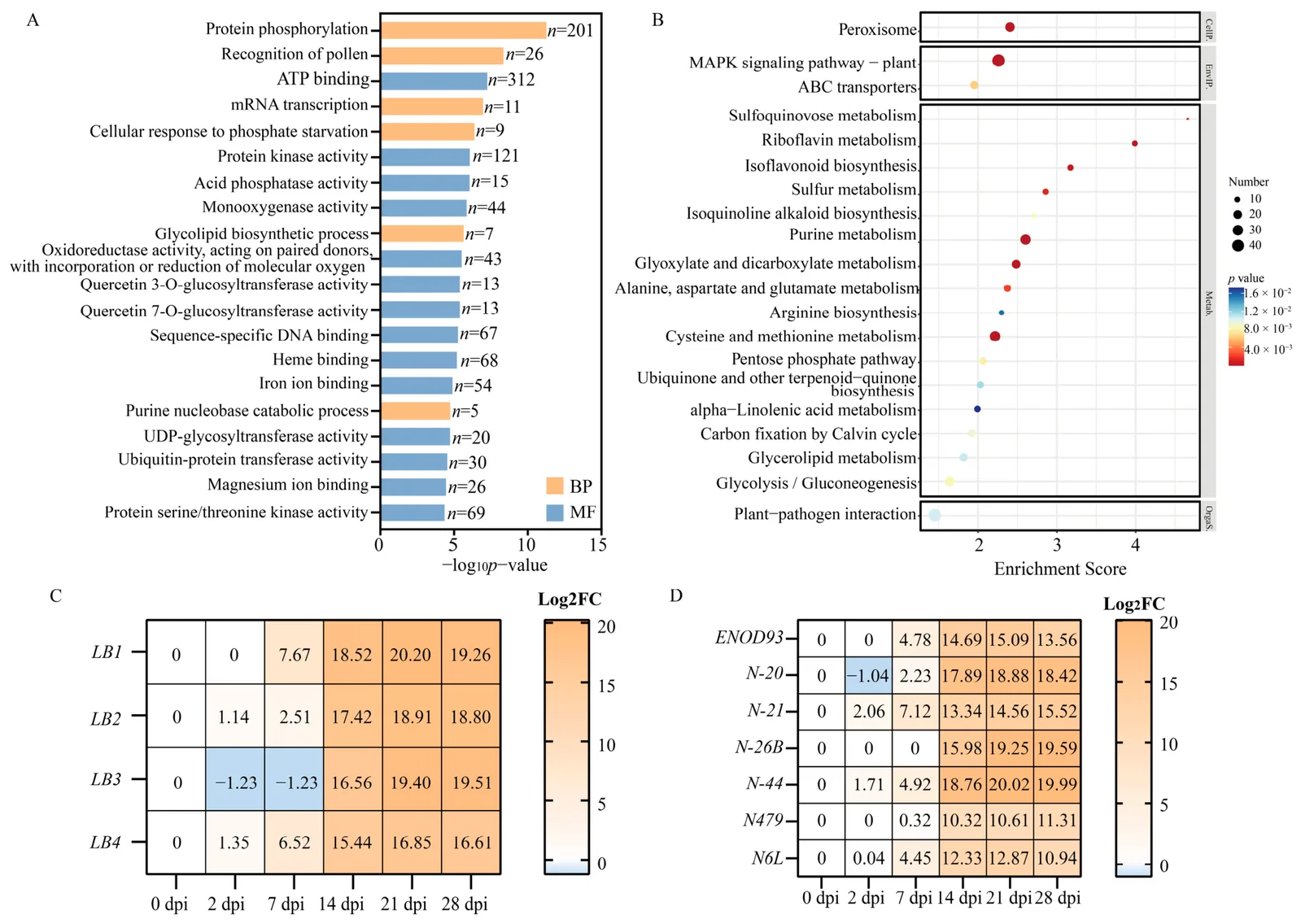
Functional enrichment analysis and symbiotic marker gene expression profiles of the continuously up-regulated Module 39. (**A**) GO enrichment analysis (top 20 significantly enriched terms, ranked by −log_10_*p*-value). Terms are categorized into biological process (BP) and molecular function (MF). N indicates the number of genes enriched in each term. (**B**) KEGG pathway enrichment analysis (top 20 significantly enriched pathways). Bubble color represents the *p*-value, bubble size indicates the number of genes annotated to the pathway, and ES denotes the enrichment score. (**C**) Heatmap showing the relative expression levels (log_2_FC relative to 0 dpi) of leghemoglobin genes across different time points. (**D**) Heatmap showing the relative expression levels (log_2_FC relative to 0 dpi) of nodulin genes across different time points.

To elucidate the biological functions of the continuously up-regulated Module 39, we performed Gene Ontology (GO) and Kyoto Encyclopedia of Genes and Genomes (KEGG) enrichment analyses on the 2732 DEGs assigned to this module. GO enrichment analysis identified the top 20 significant terms (Figure 6A), which fell into two categories: biological process and molecular function. Protein phosphorylation (GO:0006468) ranked first among the enriched biological process terms, with 201 genes annotated (−log_10_*p* = 11.27, *n* = 201). These genes set included well-known symbiotic regulators such as the receptor kinase *GmSymRKβ*, the immune repressor *EDR1*, and multiple CRK family members (Table S2). The enrichment of these signaling-related genes supports the involvement of Module 39 in symbiotic signal transduction and the fine-tuning of defense responses during symbiosis. In terms of molecular function, ATP binding (GO:0005524) topped the list with 312 annotated genes (−log_10_*p* = 7.26). This term directly supports the necessary substrate basis for protein kinase-mediated phosphorylation. Meanwhile, monooxygenase activity (GO:0004497) was also significantly enriched (−log_10_*p* = 5.86, *n* = 44) and included genes encoding key isoflavonoid biosynthesis enzymes, such as isoflavone synthase *CYP93C* and isoflavone 2′-hydroxylase *CYP81E11*. In addition, the enrichment of heme binding (GO:0020037, −log_10_*p* = 5.19, *n* = 68) and iron ion binding (GO:0005506, −log_10_*p* = 4.89, *n* = 54) further indicated a broad transcriptional activation of redox-related proteins within this module.

KEGG pathway enrichment analysis further revealed that Module 39 was significantly enriched in multiple pathways involved in metabolism and signal transduction (Figure 6B). Among the signal transduction pathways, the plant MAPK signaling pathway (gmx04016, enrichment score = 2.24) was the most significantly enriched, with 44 genes assigned. This term included multiple WRKY transcription factors (*WRKY19*, *WRKY39*, *WRKY49*, and *WRKY62*) and pathogenesis-related proteins (*PR1-6*, *PR1-7*, *PRI1-9*, etc.) (Table S3), suggesting that Module 39 genes are involved in symbiotic signal transduction and defense response regulation. Plant–pathogen interaction (gmx04626, ES = 1.43) was also significantly enriched and contained 43 genes. This pathway overlaps with the GO terms enriched for EDR1 and CRK family receptor kinases, reinforcing the view that symbiotic modulation of defense responses is tightly controlled. At the metabolic level, isoflavonoid biosynthesis (gmx00943, ES = 3.16) was significantly enriched, with 11 genes annotated. Among these were isoflavone synthase *HIDH*, isoflavone 2′-hydroxylase *I2′H/CYP81E1*, and isoflavone 7-O-glucosyltransferase *IF7GT* (Table S3), matching the GO enrichment results for monooxygenase activity and UDP-glycosyltransferase activity. Purine metabolism (gmx00230, ES = 2.59), cysteine and methionine metabolism (gmx00270, ES = 2.20), peroxisome (gmx04146, ES = 2.39), and sulfur metabolism (gmx00920, ES = 2.85) pathways were also significantly enriched, pointing to a broader involvement of Module 39 genes in primary metabolism and redox regulation. Collectively, Module 39 genes were continuously involved in signal transduction, isoflavonoid biosynthesis, and primary metabolic reprogramming during the mid-to-late stages of symbiosis in soybean roots. Notably, Module 39 also contained leghemoglobin (Figure 6C) and nodulin genes (Figure 6D). Temporal expression analysis showed that leghemoglobin and nodulin genes were barely expressed at 2–7 dpi, but surged sharply from 14 dpi onwards and reached their peak at 28 dpi (Figure S4B–L). Furthermore, we analyzed the expression changes (ΔLog_2_FC) of these symbiotic marker genes between adjacent intervals (Figure S4A). Repeated-measures one-way ANOVA followed by Tukey’s test confirmed that the magnitude of the transcriptional shift in the 7 to 14 dpi interval was significantly greater than that of all other adjacent intervals (*p* < 0.05). This temporal expression pattern coincided with the progression of nodule maturation, providing transcriptional evidence that reprogramming associated with Module 39 contributes to symbiotic nitrogen fixation.

### 2.7. GO and KEGG Enrichment Analysis of Genes in the Continuously Down-Regulated Module 8

**Figure 7:**
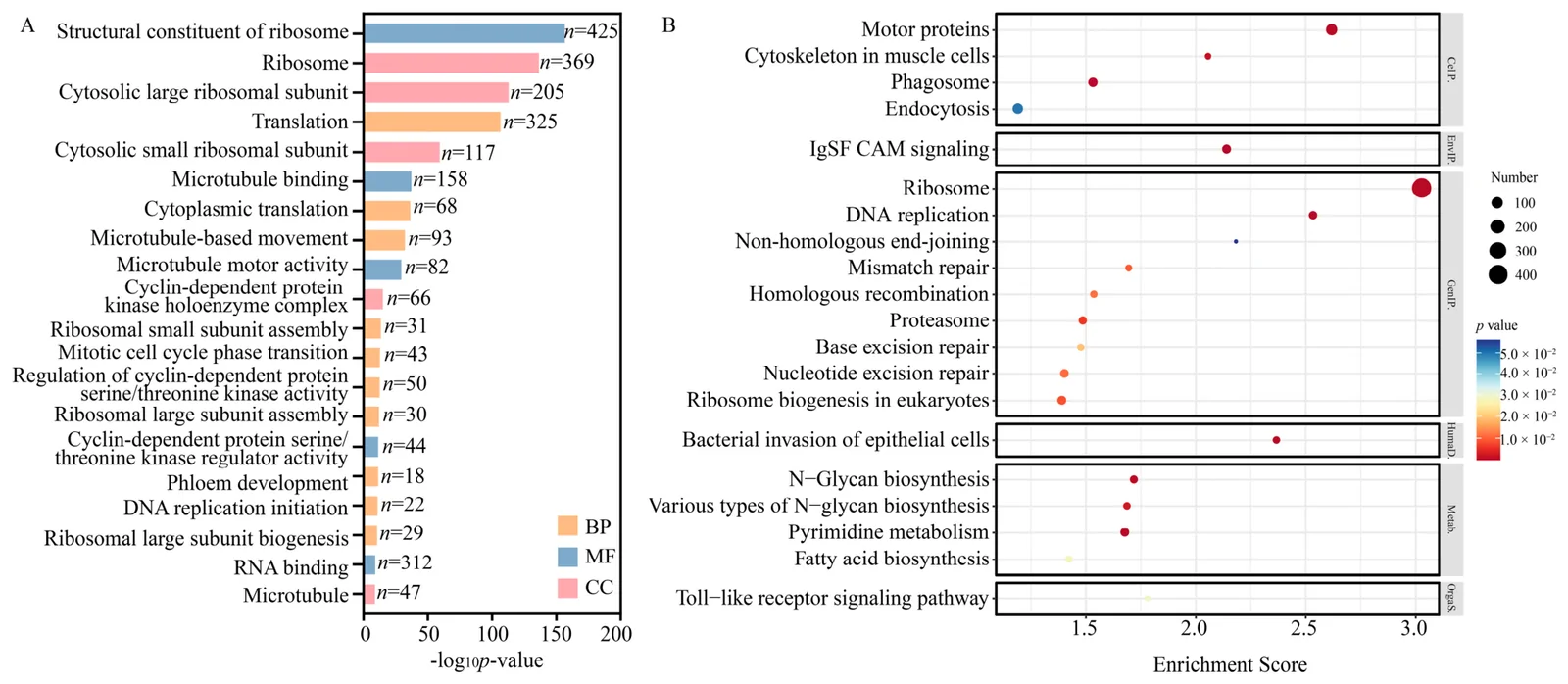
Functional enrichment analysis of the continuously down-regulated Module 8. (**A**) GO enrichment analysis (top 20 significantly enriched terms, ranked by −log_10_*p*-value). Terms are categorized into biological process (BP), molecular function (MF), and cellular component (CC). N indicates the number of genes enriched in each term. (**B**) KEGG pathway enrichment analysis (top 20 significantly enriched pathways). Bubble color represents the *p*-value, bubble size indicates the number of genes annotated to the pathway, and ES denotes the enrichment score.

To elucidate the biological functions of the continuously down-regulated Module 8, we performed GO and KEGG enrichment analyses on the 9163 DEGs assigned to this module. The top 20 GO terms (Figure 7A) and KEGG pathways (Figure 7B) by significance are shown. Genes assigned to this module were significantly enriched in GO terms related to ribosome assembly, translation, and cytoskeletal organization (Table S4). The most significant molecular function term was structural constituent of ribosome (GO:0003735, −log_10_*p* = 156.65), containing 425 genes. Ribosome (GO:0005840, −log_10_*p* = 136.43, *n* = 369), cytosolic large ribosomal subunit (GO:0022625, −log_10_*p* = 112.99, *n* = 205), and cytosolic small ribosomal subunit (GO:0022627, −log_10_*p* = 59.33, *n* = 117) were all significantly enriched at the cellular component level. The biological process terms translation (GO:0006412, −log_10_*p* = 106.64, *n* = 325) and cytoplasmic translation (GO:0002181, −log_10_*p* = 36.35, *n* = 68) were also significantly over-represented.

KEGG enrichment analysis revealed ribosome (gmx03010, ES = 3.02) as the most significantly enriched pathway (Table S5), corroborating the GO enrichment results for ribosome-related categories. These findings collectively indicate a time-dependent transcriptional suppression of genes involved in ribosome biogenesis and translation in soybean roots after rhizobial inoculation. In addition, genes in this module were also significantly enriched in cytoskeleton-related GO terms, including microtubule binding (GO:0008017, −log_10_*p* = 37.30, *n* = 158), microtubule motor activity (GO:0003777, −log_10_*p* = 29.49, *n* = 82), and microtubule-based movement (GO:0007018, −log_10_*p* = 32.22, *n* = 93). Consistent with the GO results, the KEGG pathway Motor proteins (gmx04814, ES = 2.61) was also significantly enriched, reinforcing the observation that cytoskeleton-related transcripts declined after inoculation. The GO and KEGG enrichment data collectively showed that the sustained down-regulation of Module 8 genes was enriched in ribosome assembly, translation, and cytoskeleton-related processes. These findings suggest that, as symbiosis becomes established, soybean roots progressively down-regulate multiple basal metabolic activities at the transcriptional level, thereby conserving energy for symbiotic nitrogen fixation.

### 2.8. Expression Pattern Analysis of Key Signaling and Metabolic Pathways

#### 2.8.1. Temporal Expression Patterns of Isoflavonoid Biosynthesis Genes

**Figure 8:**
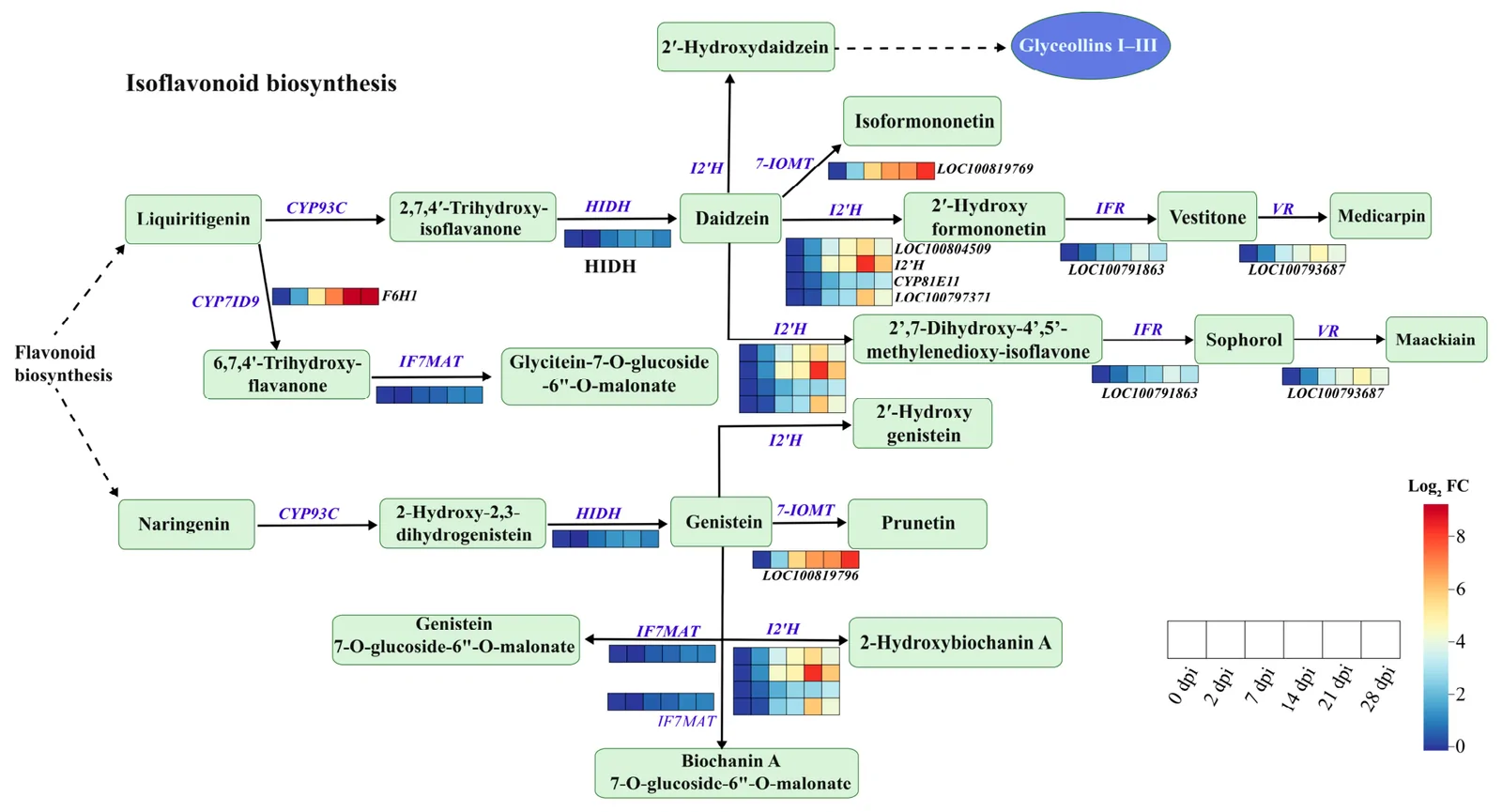
Temporal expression profiles of key genes involved in the isoflavonoid biosynthesis pathway. The left panel presents a schematic diagram of the isoflavonoid biosynthesis pathway (adapted from KEGG gmx00943). Liquiritigenin enters the isoflavonoid branch and is converted by isoflavone synthase (*CYP93C*) to daidzein and genistein. The right panel shows heatmaps of key enzyme genes at six time points (0–28 dpi), with color from blue to red representing low to high log_2_FC. Dashed arrows indicate multi-step reactions.

To characterize the temporal activation of the isoflavonoid biosynthesis pathway (gmx00943) within the continuously up-regulated Module 39, we examined the expression patterns of key enzyme genes in this pathway (Figure 8). The isoflavonoid biosynthesis pathway draws from phenylpropanoid metabolism. Liquiritigenin enters the isoflavonoid branch and is converted by isoflavone synthase (*CYP93C*) into daidzein and genistein. These are further modified through hydroxylation, glycosylation, and malonylation to produce various derivatives. Among these genes, the flavanone 6-hydroxylase gene *F6H1* (*CYP71D9*) showed the largest increase, rising from log_2_FC 1.61 at 2 dpi to 9.24 at 28 dpi (Table S6). This suggests that hydroxylation of isoflavonoid precursors was substantially enhanced during the mid-to-late symbiotic stages.

2-hydroxyisoflavanone dehydratase *HIDH* showed a slight decrease at 2 dpi, then gradually increased, peaking at 1.48 at 21 dpi. *I2′H* peaked at log_2_FC 8.31 at 21 dpi (Table S6), roughly 2.6 times the peak of *CYP81E11* (log_2_FC 3.19). This suggests that *I2′H* is the primary gene responsible for converting daidzein to 2′-hydroxydaidzein. In addition, *7-IOMT* (*LOC100819796*) rose sharply and continuously (peak log_2_FC 8.29 at 28 dpi) (Table S6), indicating a marked enhancement of the methylation branch during the mid-to-late symbiotic stages. Isoflavone 7-O-glucosyltransferase *IF7GT* peaked at log_2_FC 4.29 at 21 dpi. However, the isoflavone 7-O-glucoside-6″-O-malonyltransferase gene (*IF7MaT*) showed only a slight increase, with a log_2_FC of 1.03 at 28 dpi (Table S6). This indicates that glycosylation was activated during the mid-to-late symbiotic stages, while the transcriptional response of malonylation remained limited. In summary, the transcriptomic data revealed a continuous activation of the isoflavonoid biosynthesis pathway during the mid-to-late symbiotic stages after HK601 inoculation, particularly characterized by the pronounced up-regulation of genes involved in the hydroxylation (*F6H1*, *I2′H*) and methylation (*7-IOMT*) branches.

#### 2.8.2. Temporal Expression Patterns of Plant MAPK Signaling Pathway Genes

**Figure 9:**
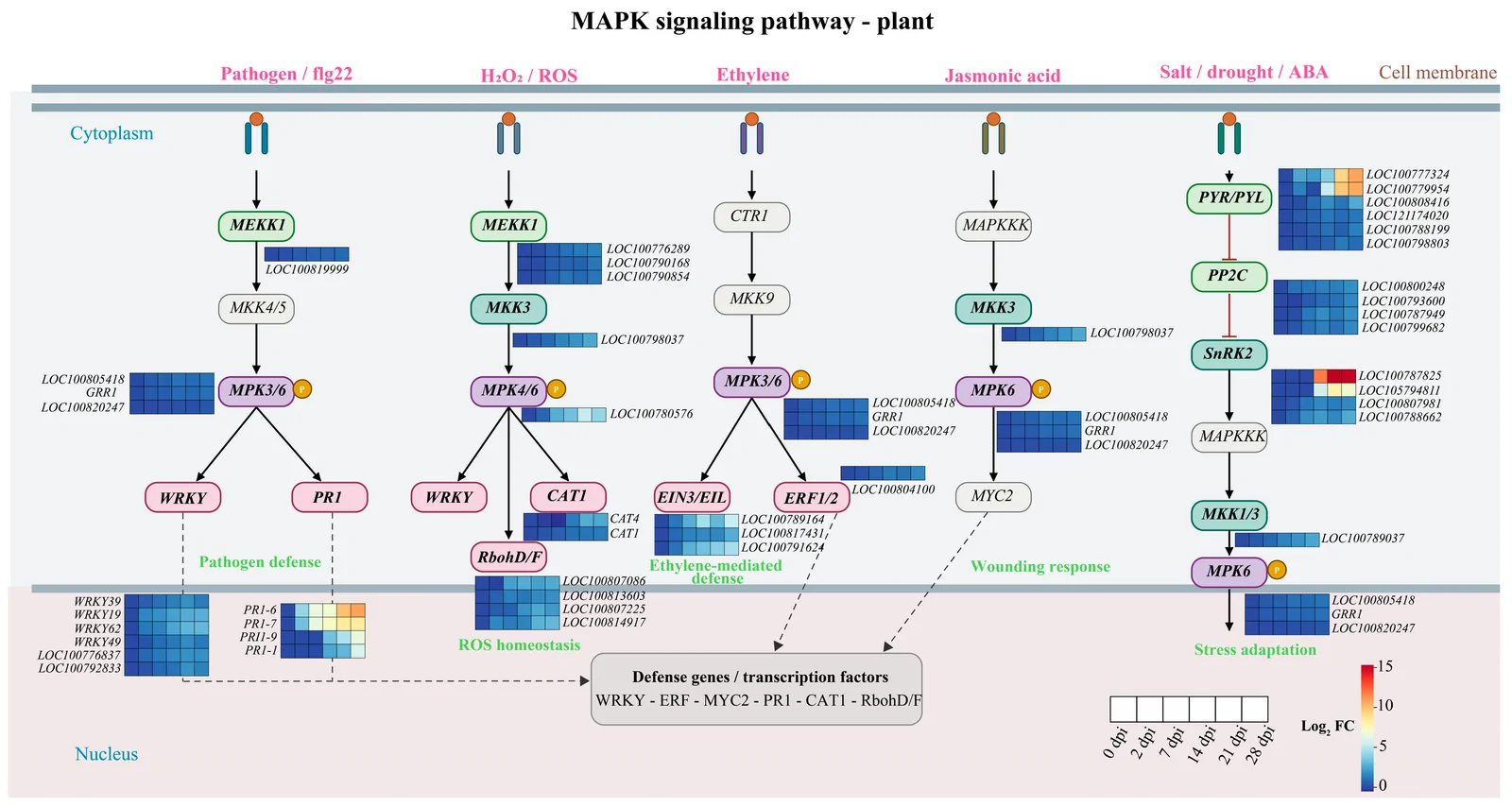
Temporal expression profiles of key genes in the plant MAPK signaling pathway. The left panel shows a schematic diagram of the pathway with genes arranged by signal transduction direction. The right panel shows heatmaps of gene expression at six time points (0–28 dpi), with color from blue to red indicating low to high log_2_FC.

A temporal expression analysis was performed on the 44 genes enriched in the plant MAPK signaling pathway (gmx04016) within Module 39. Under the KEGG framework, these genes mapped to a complete signaling cascade extending from upstream signal perception to downstream defense output (Figure 9). The 44 genes exhibited a time-dependent increase in expression, with up-regulation beginning at 2 dpi, a substantial rise after 14 dpi, and sustained high levels through 21–28 dpi (Table S7). However, the 44 genes did not show coordinated up-regulation; the timing of their response varied with their positions along the pathway. *LOC100777324* and *LOC100779954* (PYL family members) exhibited the strongest up-regulation among the ABA receptor genes, with log_2_FC of 10.81 and 10.67 at 28 dpi (Table S7). These results point to activation of ABA signaling in the mid-to-late symbiotic period, possibly contributing to root adaptive responses following inoculation. SnRK2 kinase *LOC100787825* showed little change before 14 dpi (log_2_FC near 0), then rose sharply to 11.82 at 14 dpi and peaked at 15.34 at 21 dpi (Table S7). This exhibited a typical “switch-like” activation pattern. Another SnRK2 family member (*LOC106794811*) followed a similar trend, though with a lower amplitude (peak log_2_FC 7.96). During the mid-to-late symbiotic stages, *PYR*/*PYL* and *SnRK2* were activated one after the other. This matched the signal flow in the pathway map, from ABA perception to kinase activation. This temporal coupling points to cross-talk between ABA signaling and the MAPK pathway during this period.

WRKY transcription factors were also consistently up-regulated. *WRKY62* and *WRKY19* peaked at log_2_FC 3.00 and 2.69, respectively (Table S7). This suggests that after rhizobial inoculation, MAPK signaling in soybean roots was relayed to the transcriptional regulation level, driving the expression of defense-related genes. PR1 family genes were markedly up-regulated, with *PR1-6* and *PR1-7* peaking at log_2_FC 10.95 and 8.45, respectively (Table S7). This further supports that the signal had reached the downstream defense response level. *RbohD* and *CAT* were also up-regulated in parallel, acting in the ROS production and scavenging branches, respectively (Table S7). This suggests a coordinated regulation of ROS homeostasis during the mid-to-late symbiotic stages in soybean roots. *EIN3*/*EIL* was also up-regulated during the mid-to-late symbiotic stages (Table S7), suggesting that ethylene signaling may also participate in the root response during this period. Together, these results indicate that after rhizobial inoculation, soybean roots simultaneously engaged multiple downstream processes through the MAPK pathway, including hormone signaling, immune/defense responses, and ROS homeostasis regulation. This occurred mainly after 14 dpi, coinciding with the window of nodule maturation, and may function to maintain the defense–symbiosis balance during the later symbiotic stages.

### 2.9. Transcript-Level Analysis and mRNA Validation by qRT-PCR

**Figure 10:**
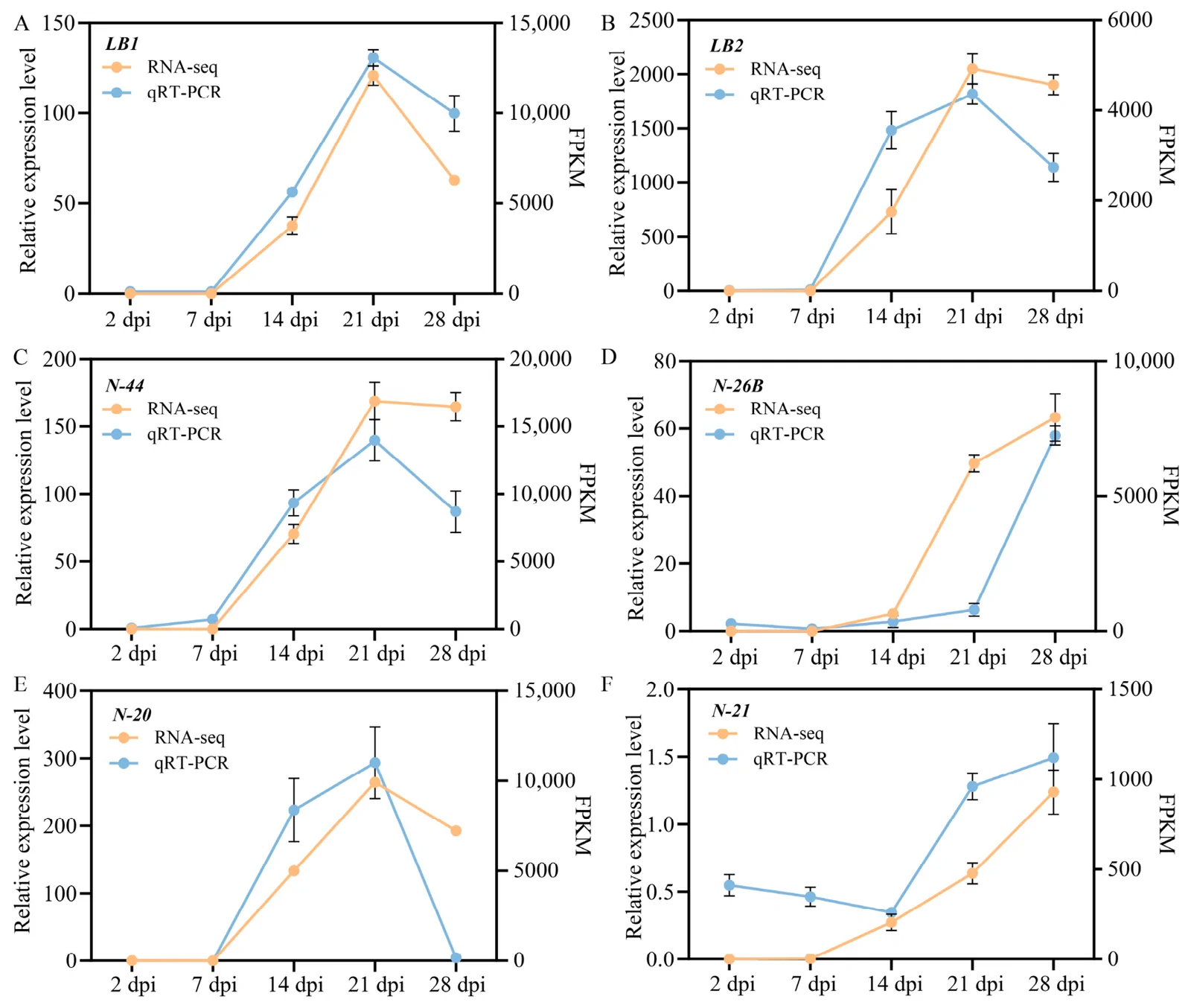
qRT-PCR validation of transcriptomic data. (**A**–**F**) Relative expression levels and FPKM values of the DEGs *LB1*, *LB2*, *N-44*, *N-26B*, *N-20*, and *N-21*, respectively. The left and right Y-axes represent the relative expression levels and RNA-seq FPKM values of the DEGs (mean ± SD, *n* = 3 biological replicates), respectively.

To assess the robustness of the transcriptome data, we selected two leghemoglobin genes (*LB1* and *LB2*) and four nodulin genes (*N-44*, *N-26B*, *N-20*, and *N-21*) for qRT-PCR analysis (Figure 10). The qRT-PCR results showed expression trends consistent with the transcriptome analysis, confirming the accuracy of the RNA-seq data.

## 3. Discussion

Rhizobia–soybean compatibility largely determines nodulation efficiency. Rhizobia are often highly host-specific. Most strains form effective symbioses with only a few soybean cultivars [3]. In this study, *B. ottawaense* HK601 nodulated Williams 82 and 11 soybean cultivars from different accumulated temperature zones in Heilongjiang, China. Nodulation efficiency varied among cultivars, but overall HK601 showed broad-spectrum nodulation. This may be attributed to the inherent broad-host-range property of *B. ottawaense*. Its nodulation genes (nod genes) are highly conserved [25], and the encoded nod factors can recognize flavonoid signals secreted by roots of diverse soybean cultivars [26]. On the other hand, *B. ottawaense* HK601 was originally isolated from the root nodules of the soybean cultivar ‘Heike 60’ in Heilongjiang Province, China. *B. ottawaense* HK601 has undergone long-term co-evolution with local soybean cultivars. Its nodulation-related genes are well matched to the signaling molecules of these local cultivars, which may help it overcome cultivar differences. As a result, HK601 may have advantages in cultivar compatibility, environmental adaptation, and competition against indigenous rhizobia [27].

Soybean nodule development proceeds in a defined order. Rhizobia infect root hairs and form infection threads at 0–2 dpi [28]. Cortical cell divisions produce nodule primordia and meristems around 7 dpi [29,30]. Nodules then mature and start fixing nitrogen at 14–21 dpi, with leghemoglobin accumulation and nitrogenase activation [31]. By 28 dpi, nitrogen fixation levels off [23]. In this study, soybean roots inoculated with *B. ottawaense* HK601 were collected at six time points (0, 2, 7, 14, 21, and 28 dpi) for transcriptome sequencing, based on the temporal framework of nodule development described above. The aim was to characterize the transcriptional changes in soybeans during the symbiotic process. Temporal transcriptomic analysis revealed that the number of DEGs increased from 3858 at 2 dpi to 15,235 at 28 dpi. This upward trend coincided with the progression of nodule developmental stages. At 7 dpi, up-regulated DEGs were about twice as many as down-regulated ones. By 14 dpi, the up/down ratio dropped to about 1.10:1, and leghemoglobin and nodulin genes began to rise sharply. This indicates that 14 dpi is a key stage in the transition from nodule structural establishment to nitrogen fixation. This is consistent with Fan et al., who found that nodule-specific genes are progressively activated during development [32].

In this study, we focused on two key modules: the continuously up-regulated Module 39 and the continuously down-regulated Module 8. In Module 39, GO enrichment analysis identified protein phosphorylation as the most significantly enriched term, encompassing *SYMRK-BETA*, *EDR1*, and multiple CRK family receptor kinases. *SYMRK* functions as a core co-receptor in the nodulation signaling pathway, and its activity is dependent on a phosphorylation cascade. The study has demonstrated that *SYMRK* transmits extracellular signals into the cell through phosphorylation [16]. In *Arabidopsis*, *EDR1* encodes a Raf-like MAPKKK that negatively regulates immune signaling, as its loss-of-function results in the hyperactivation of defense responses [33]. CRK family members, as cysteine-rich receptor-like kinases, are important components of plant immune signal perception and transduction [34]. They participate in immune signaling by regulating Ca^2+^ influx, ROS production, and MAPK cascade activation [35]. The simultaneous up-regulation of these distinct protein kinases indicates sustained activation of the phosphorylation signaling network during the mid-to-late symbiotic stages, involving coordination among multiple functional nodes. KEGG enrichment of the MAPK signaling pathway further confirms the continuous operation of the phosphorylation cascade. Sun et al. [36] constructed a single-nucleus transcriptomic atlas of soybean roots and nodules. They found that protein kinase-related genes were enriched in nodule cells. This agrees with our observation that phosphorylation signaling-related genes were continuously up-regulated. Module 39 contained both *EDR1* (an immune negative regulator) and the CRK family (positive regulatory receptor kinases). *EDR1* limits defense overactivation [33], while CRK family members initiate and transduce immune signals [35]. Both were up-regulated together at the transcriptional level. This suggests a finely tuned defense regulatory mechanism in roots during the mid-to-late symbiotic stages.

Relative to 0 dpi, DEGs in Module 8 declined from 14 to 28 dpi after rhizobial inoculation. They were enriched in basal processes such as ribosome assembly, translation, and cytoskeletal organization. The most significant term was ribosome structural constituent (GO:0003735). Genes for ribosomal components and the cytoskeleton were highly expressed at 2–7 dpi (the nodule primordium formation stage), likely supporting cortical cell division, meristem establishment, and cellular structural reorganization during infection. After 14 dpi, as nodules entered the nitrogen fixation stage, meristematic activity declined, and gene expression levels correspondingly decreased. This temporal pattern is consistent with what DelPercio et al. observed in soybean nodules, where ribosome biogenesis-related genes were enriched early in development (2–3 wpi) [23]. Cytoskeletal proteins have been shown to function in legume nodulation across multiple species. Hlavackova et al. reviewed the role of the cytoskeleton in legume–rhizobia symbiosis and emphasized that dynamic rearrangements of microtubules and actin filaments are central to rhizobial infection and nodule development [37]. In *Medicago truncatula*, microtubule-associated proteins rearrange markedly during infection thread formation and nodule primordium development [38]. The actin cytoskeleton plays a critical role in infection thread elongation and symbiosome release [39]. These studies show that cytoskeletal dynamics are central to cell morphogenesis and infection progression during early nodule development. In this study, genes related to microtubule binding were highly expressed early in symbiosis and then down-regulated. This decrease indicates a lower demand for protein synthesis to maintain routine cytoskeletal dynamics in roots during the mid-to-late symbiotic stages. Such a reduction may support nodule development and the establishment of nitrogen fixation. Overall, genes in Module 39 were persistently up-regulated, whereas genes in Module 8 were persistently down-regulated. These two patterns occurred synchronously and were functionally complementary. Down-regulation of Module 8 reduced the demand for transcriptional and translational resources, thereby freeing resources for the establishment of symbiotic nitrogen fixation. Activation of Module 39, in turn, used these resources to support defense metabolism and maintain symbiotic functions. This pattern may reflect how the host reallocates limited resources during symbiosis establishment.

Isoflavonoid biosynthesis plays dual roles in legume–rhizobia symbiosis. On one hand, isoflavonoids act as signaling molecules that induce the expression of rhizobial nodulation genes and initiate the establishment of symbiosis [40,41]. On the other hand, their derivatives function as phytoalexins and participate in defense responses [42,43]. In this study, the isoflavonoid biosynthesis pathway in soybean roots was activated in a temporally cascaded manner after HK601 inoculation. Different modification branches exhibited marked differences in both expression intensity and temporal dynamics. Among cytochrome *P450* genes, *F6H1* (*CYP71D9*) showed the largest up-regulation. Its expression increased from log_2_FC 1.61 at 2 dpi to 9.24 at 28 dpi. This suggests that hydroxylation of isoflavonoid precursors was greatly enhanced during mid-to-late symbiosis. The isoflavone 2′-hydroxylase gene *I2′H* reached a peak log_2_FC of 8.31 at 21 dpi, which was significantly higher than that of *CYP81E11* (peak log_2_FC 3.19) within the same family. *I2′H* belongs to the *CYP81E* subfamily and catalyzes the 2′-hydroxylation of the isoflavone B-ring, a modification that is essential for the biosynthesis of pterocarpan phytoalexins, such as glyceollin in soybean [44,45]. Lin et al. performed a comparative transcriptomic analysis of *B. ottawaense* Bott 59 and *B. diazoefficiens* USDA110 at 3 dpi [24]. They found that Bott 59 specifically induced the expression of isoflavonoid biosynthesis genes, including *I2′H*, at the early stage [24]. In this study, *I2′H* also showed sustained and strong up-regulation from 14 to 28 dpi. This suggests that the regulation of the hydroxylation branch by *B. ottawaense* remains persistently active during the mid-to-late symbiotic stages. Given the dual functions of isoflavonoids, the sustained high expression of *F6H1* and *I2′H* during mid-to-late symbiosis represents an unresolved metabolic flux. Because transcriptomic data only reflect gene expression levels rather than actual metabolic fluxes, the available data cannot distinguish whether this flux contributes to sustained symbiotic signaling or to the synthesis of the defensive phytoalexin glyceollin. The gene *7-IOMT* exhibited a sustained upward trend, reaching log_2_FC 8.29 at 28 dpi, indicating robust activation of the methylation branch during the mid-to-late symbiotic stages. *7-IOMT* methylates the 7-hydroxyl group on the A-ring of isoflavones [46]. This modification increases their hydrophobicity and stability [47,48]. Some methylated derivatives, such as formononetin and biochanin A, participate in chemical communication and stress responses during plant–microbe interactions [49,50]. The hydroxylation branch (*I2′H*) and methylation branch (*7-IOMT*) both increased steadily during the mid-to-late symbiotic stages. This indicates a potential coordinated activation of isoflavonoid skeleton hydroxylation and methylation after skeleton formation. However, based on current transcriptomic data, it remains unclear whether these branches compete for substrates or how metabolic fluxes are allocated over time. The specific functions of methylated derivatives in nodule symbiosis also require further investigation. Future studies integrating comprehensive metabolite profiling and enzymatic assays are required to address these gaps and elucidate their metabolic fate.

The MAPK pathway functions in a time-dependent manner during symbiosis. Early in symbiosis, nod factors rapidly activate specific MAPK cascades. These cascades contribute to root hair deformation, infection thread formation, and initial transduction of early nodulation signals [51,52]. However, this early transient activation also acts as a negative regulator to limit excessive infection [53]. ABA is an important upstream hormone signal in this pathway. Its inhibitory effect on early nodulation in legumes is well documented [53,54]. Exogenous ABA application has been shown to inhibit nodule formation in *Pisum sativum*, *G. max*, *L. japonicus*, *M. truncatula*, and *Phaseolus vulgaris*. This inhibition involves several mechanisms, including reduced rhizobial numbers, suppressed root hair infection, and blocked cortical cell division [54,55,56]. However, in this study, ABA signaling was significantly activated only during the mid-to-late symbiotic stages (14–28 dpi), suggesting that its function may shift from early inhibition to adaptive regulation for maintaining symbiotic homeostasis at later stages. In the downstream effector region, the sustained up-regulation of WRKY transcription factors indicated that the MAPK signal had been relayed to the level of transcriptional regulation. WRKY transcription factors act as key transcriptional hubs in plant immune signaling and can bind to the *PR1* promoter to activate its transcription during symbiotic interactions [57]. Consistently, the pronounced up-regulation of *PR1-6* and *PR1-7* in this study confirmed that the MAPK signal had extended to downstream defense responses. Regarding ROS regulation, *RbohD* and *CAT* were up-regulated in parallel, acting in the production and scavenging branches of ROS, respectively. *RBOH* family members play a positive role in maintaining nodule function. In *M. truncatula*, *MtRbohA* is significantly up-regulated in functional nodules, and its RNAi silencing reduces nitrogen fixation capacity, indicating that appropriate levels of ROS are essential for nodule development and functional maintenance [58]. However, excessive accumulation of ROS can cause oxidative damage to nodules. In *L. japonicus* leghemoglobin-deficient mutants, ROS accumulation in nodules was accompanied by premature nodule senescence. *LjGPX1* and *LjGPX3* are highly expressed in the infected zone of nodules to prevent lipid peroxidation. These findings indicate that the antioxidant system is essential for maintaining the integrity of nodule cells. Thus, the coordinated up-regulation of *RbohD* and *CAT* in this study may reflect an active regulatory mechanism for maintaining ROS homeostasis in soybean roots during the mid-to-late symbiotic stages. This suggests that *RBOH*-mediated ROS production may support nodule function. At the same time, increased CAT activity could prevent cellular damage caused by excessive ROS.

This study revealed the transcriptional reprogramming dynamics in symbiotic soybean roots. However, some limitations remain. First, the transcriptomic analysis used the 0 dpi point (the time of inoculation) as the baseline, and no parallel non-inoculated control was sequenced. Therefore, the observed changes in DEGs mainly reflect temporal dynamics after inoculation, rather than gene expression specifically induced by rhizobia; future studies should include non-inoculated controls per time point. Second, transcriptomic data came from middle root segments. These data could not distinguish the transcriptional responses of nodule tissues from those of other root parts. In addition, the biological functions of the key genes and the actual accumulation of isoflavonoid metabolites remain to be further validated through future metabolomic and functional assays.

## 4. Materials and Methods

### 4.1. Plant Materials

Thirteen soybean (*G. max* L.) cultivars, namely Heike60 (HK60), Williams82 (WS82), Heihe43 (HH43), Heike86 (HK86), Qinong7 (QN7), Qinong12 (QN12), Qinong26 (QN26), Nongqingdou212 (NQD212), Nongqingdou103 (NQD103), Kangxianchong8 (KXC8), Mudou11 (MD11), Mudou36 (MD36), and Dongsheng79 (DS79), were kindly provided by the Heilongjiang Academy of Agricultural Sciences (Harbin, China). Among these, HK60 was used for symbiotic phenotype evaluation (including nodulation) and time-series transcriptome sequencing analysis, while the remaining 12 cultivars were used for the broad-spectrum nodulation assay of HK601.

### 4.2. Isolation and Identification of Rhizobia

Fresh root nodules were collected from the rhizosphere of HK60 soybeans, washed with clean water, disinfected on the surface with a 1% sodium hypochlorite solution for 5 min, and rinsed 5–6 times with sterile water. Sterilized nodules were crushed, and those with pinkish-red sections were selected for streaking on Congo red-containing YMA medium. A single colony with neat edges, a slightly raised center, a translucent milky-white color, and a mucous, viscous consistency was isolated and purified.

DNA was extracted using the TIANamp Bacteria DNA Kit (Tiangen, Beijing, China), and PCR amplification and sequencing were performed using the primers listed in Table S8 for the *16S rDNA*, *glnII*, *atpD*, and *recA* [59,60]. The resulting sequences were then aligned against the national center for biotechnology information (NCBI) database using BLAST (https://blast.ncbi.nlm.nih.gov/Blast.cgi, accessed on 15 October 2025) to obtain reference sequences. All reference strain names used for phylogenetic analysis and GenBank accession numbers are listed in Table S9. The partial sequences of strain HK601 were submitted to the GenBank and GenBank accession numbers were obtained (Table S9). Multiple sequence alignment was performed using MEGA 7, followed by manual trimming. The four genes were concatenated into a combined sequence using Sequence Matrix v1.7.8. Phylogenetic trees were reconstructed using both maximum likelihood (ML) and Bayesian inference (BI) methods based on concatenated gene sequences (*16S rDNA* + *glnII* + *atpD* + *recA*). The ML analysis was performed using IQTree v3.0.1 [61] with 1000 bootstrap repetitions; the BI analysis was performed using MrBayes v3.2.7 [62] under the GTR+G+I model (selected by jModelTest v2.1.10), with 2,000,000 MCMC iterations and a 25% burn-in. Nodal support was evaluated by bootstrap values (ML) and posterior probabilities (BI).

### 4.3. Gram Staining

First, 1 drop of saline solution was added to the center of a clean microscope slide, then a bacterial colony was picked up and spread evenly into a thin layer approximately 1 cm in diameter. After air-drying, the microscope slide was held over the flame of an alcohol burner to fix the morphology of the bacterial cells. Next, crystal violet was added dropwise and evenly; after staining for 1 min, this was rinsed thoroughly with sterile water. A drop of Lugol’s iodine solution was added and allowed to act as a mordant for 1–2 min; it was then rinsed with sterile water and spin-dried. A drop of 95% ethanol was added to decolorize for 30 s; then it was rinsed thoroughly with sterile water to stop the decolorization process, and the color of the bacterial cells was observed under a 100× optical microscope.

### 4.4. Pot Culture and Nodulation Assay

Vermiculite and perlite were mixed (*v*/*v* = 2:1) and autoclaved at 121 °C for 60 min. Selected soybean seeds with smooth and intact coats were surface-sterilized with 1% NaClO, rinsed with sterile water, and directly sown in the sterilized substrate in pots (11 cm upper diameter × 12 cm height) under a 16/8 h light/dark photoperiod provided by four 36 W LED light strips. After the first true leaf fully expanded (approximately 7 days after sowing), rhizobial cells were harvested by centrifugation and resuspended in sterile water to an OD_600_ value of 0.8–1.0. Each seedling received 1 mL of rhizobial suspension at the root zone, whereas the control group received an equal volume of sterile water and remained uninoculated. Soybean plants were incubated at 28 °C for 28 days, with daily observation of growth and periodic supplementation of nitrogen-free Hoagland’s nutrient solution. At 28 dpi, relative leaf chlorophyll content was measured as SPAD values using a Jinkelida TYS-4N portable chlorophyll meter (Beijing Jinkelida Electronic Technology Co., Ltd., Beijing, China). The instrument also provides an estimated nitrogen content (in mg/g) based on a precalibrated model. These estimated values were used as an auxiliary indicator of leaf nitrogen status. The shoot height and root length were measured using the base of the stem as the starting point. At the same time, agronomic traits such as the number of effective nodules, fresh weight of effective nodules, dry weight of effective nodules, shoot fresh weight, root fresh weight, shoot dry weight, and root dry weight were measured.

### 4.5. Determination of Total Nitrogen Content

Plant samples were oven-dried at 105 °C for 30 min to inactivate enzymatic activity and then dried at 80 °C to constant weight. Ground samples (60-mesh) were accurately weighed at 0.2 g (accurate to 0.0001 g) into digestion tubes, mixed with 3.2 g of catalyst (K_2_SO_4_:CuSO_4_ = 15:1), and 5 mL of concentrated H_2_SO_4_ was added slowly. The digestion tubes were placed on a digestion block (GD60S, APL Technology Group (Chengdu) Co., Ltd., Chengdu, China) and heated at 200 °C initially. The temperature was maintained at 200 °C until the sample was completely charred and frothing ceased. Subsequently, the temperature was increased to 420 °C and maintained until the digest became clear and was boiling gently. Digestion was terminated when the solution became clear and blue-green. After cooling, the total nitrogen content was determined using a fully automatic Kjeldahl nitrogen analyzer (Hanon K1100, Hanon Instruments, Jinan, China).

### 4.6. RNA Isolation and Library Preparation

To ensure consistency in tissue sampling across different seedling ages, a distance-based strategy was applied using the stem base as a fixed anatomical reference point. The middle root segments (2 to 4 cm below the stem base, including the primary root, lateral roots, and root hairs, with the root tips removed) were collected at 0, 2, 7, 14, 21, and 28 dpi. The root samples were thoroughly rinsed with sterile water to remove adhering substrate, blotted dry with sterile filter paper, immediately frozen in liquid nitrogen, and stored at −80 °C. Three replicates were included for each treatment. Total RNA was extracted using TRIzol^®^ reagent (Invitrogen, Carlsbad, CA, USA) according to the manufacturer’s protocol. RNA purity and quantification were evaluated using the NanoDrop 2000 spectrophotometer (Thermo Scientific, Waltham, MA, USA). RNA integrity was assessed using the Agilent 2100 Bioanalyzer (Agilent Technologies, Santa Clara, CA, USA). Then the libraries were constructed using VAHTS Universal V10 RNA-seq Library Prep Kit (Premixed Version) according to the manufacturer’s instructions. The transcriptome sequencing and analysis were conducted by OE Biotech Co., Ltd. (Shanghai, China).

### 4.7. RNA Sequencing and Differentially Expressed Gene Analysis

The libraries were sequenced on an Illumina NovaSeq 6000 platform, and 150 bp paired-end reads were generated. Raw reads of fastq format were first processed using fastp (v0.20.1), and the low-quality reads were removed to obtain the clean reads [63]. The clean reads were mapped to the soybean Williams 82 reference genome (Wm82.a4.v1, GCF_000004515.6, NCBI) using HISAT2 (v2.1.0) [64]. FPKM [65] of each gene was calculated, and the read counts of each gene were obtained by HTSeq-count (v0.11.2) [66]. Differential expression analysis was performed using the DESeq2 (v1.22.2) [67]. A *q*-value < 0.05 and |log_2_FC| > 1 was set as the threshold for significantly differential expression genes (DEGs). Hierarchical cluster analysis of DEGs was performed using R (v3.5.1) to demonstrate the expression pattern of genes in different groups and samples.

### 4.8. Temporal Expression Pattern and Functional Enrichment Analyses

Differentially expressed genes were identified by TCC analysis of gene expression data from soybean roots at 0, 2, 7, 14, 21, and 28 dpi with rhizobia. These genes were then subjected to trend analysis using STEM (Short Time-series Expression Miner) software (version 1.3.13) [68]. The expression values of all genes were first normalized to the expression level at 0 dpi. After profile construction, gene expression data were transformed and compared with each profile. Each gene was assigned to the profile showing the highest similarity. Random permutation of time points was performed to calculate the expected gene count per profile. The significance of each profile was assessed using the hypergeometric distribution (*p* < 0.05). Modules with a False Discovery Rate (FDR)-adjusted *p*-value < 0.05 were considered significantly enriched. The temporal expression trends of these significant modules were then visualized. GO [69] and KEGG [70] enrichment analyses of genes in significant modules were performed using clusterProfiler (v3.10.0) based on the hypergeometric test. The top 20 enriched terms were visualized as bar plots and bubble plots.

### 4.9. Validation of RNA-Seq Data by qRT-PCR

To verify the transcriptome data, selected DEGs were validated by qRT-PCR. qRT-PCR primers were designed and synthesized by Sangon Biotech (Shanghai, China) (Table S10). First-strand cDNA was synthesized from soybean root RNA using a cDNA synthesis kit (Simgen, Hangzhou, China) according to the manufacturer’s protocol. qRT-PCR was performed using the Blaze Taq™SYBR^®^ Green qPCR Mix 2.0 kit (GeneCopoeia, Rockville, MD, USA), with cDNA as the template and the soybean *GmActin* gene as the internal reference gene. The 10 µL qRT-PCR reaction contained 500 ng cDNA, 2 µL primers, 2 µL SYBR Mix, and 0.4 µL ROX. Cycling conditions were 95 °C for 30 s, then 40 cycles of 95 °C for 10 s and 60 °C for 30 s, with a melting curve analysis. Relative gene expression was calculated by the 2^−ΔΔCt^ [71] method and compared with RNA-seq data to confirm the transcriptome accuracy.

### 4.10. Statistical Analysis

Data were recorded in Excel 2016, analyzed with SPSS 24 (ANOVA, *p* < 0.05 and *p* < 0.01), plotted with GraphPad Prism 8, and assembled using Adobe Illustrator 2023. For the transcriptomic data, to evaluate the temporal dynamics of symbiotic marker genes, Log_2_FC values were calculated from the FPKM values using the 0 dpi expression level as the baseline. The adjacent interval expression changes (ΔLog_2_FC) were then computed. Because destructive sampling was used, independent soybean roots were collected at different time points; therefore, individual genes were treated as paired statistical units to control for inherent baseline differences. A repeated-measures one-way ANOVA followed by Tukey’s multiple comparisons test was performed to compare the magnitude of changes across different time intervals. Statistical analyses and graph plotting were performed using GraphPad Prism 8, and figures were assembled using Adobe Illustrator 2023.

## 5. Conclusions

A broad-spectrum nodulating slow-growing rhizobium, *B. ottawaense* HK601, was isolated and identified from root nodules of the soybean cultivar ‘Heike 60’ in Heilongjiang Province, China. Pot experiments showed that it significantly promoted soybean nodulation and growth and increased plant nitrogen content. This strain provides a promising candidate resource for soybean production in cold regions. Time-series transcriptomic analysis at six time points revealed that soybean roots underwent transcriptional reprogramming after HK601 inoculation, with 14 dpi as a key turning point. Before 14 dpi, DEGs involved in signal perception and nodule structural development were actively expressed in the roots. After 14 dpi, DEGs for ribosome assembly and translation were down-regulated. In contrast, pathways for isoflavonoid biosynthesis, MAPK signaling, and defense responses were up-regulated. These changes were functionally complementary. They suggest a reallocation of transcriptional resources from basal metabolism to symbiosis-related functions in the roots. The temporally cascaded activation of isoflavonoid biosynthesis and MAPK signaling during the mid-to-late symbiotic stages may reflect their potential dual role in symbiotic signaling and defense, thereby contributing to symbiotic homeostasis. The sharp up-regulation of nodulin and leghemoglobin genes after 14 dpi provided transcriptional evidence for the establishment of symbiotic nitrogen fixation. This study offers a new transcriptomic perspective on the temporal regulation of host–rhizobia interactions and supports the application of *B. ottawaense* HK601 as a broad-spectrum rhizobial inoculant.

## Data Availability

The raw RNA-seq data have been deposited in the NCBI Sequence Read Archive (SRA) under BioProject accession PRJNA1533573. These data are currently under embargo and will be released upon publication. All other data supporting the findings of this study are included within the article and its Supplementary Materials. Further inquiries can be directed to the corresponding authors.

## Supplementary Materials

The following supporting information can be downloaded at https://www.mdpi.com/article/10.3390/plants15213420/s1: Figure S1: Broad-spectrum nodulation phenotype of *B. ottawaense* HK601 in pot experiments; Figure S2: Symbiotic compatibility analysis of *B. ottawaense* HK601 with 12 different soybean cultivars; Figure S3: Trend clustering heatmap of differentially expressed genes; Figure S4: Temporal expression dynamics and statistical validation of symbiotic marker genes across all time points. Table S1: Summary of RNA-seq data quality and alignment statistics; Table S2: GO enrichment analysis of genes in Module 39; Table S3: KEGG enrichment analysis of genes in Module 39; Table S4: GO enrichment analysis of genes in Module 8; Table S5: KEGG enrichment analysis of genes in Module 8; Table S6: Expression levels of genes enriched in the isoflavonoid biosynthesis pathway; Table S7: Expression levels of genes enriched in the plant MAPK signaling pathway. Table S8: Primers for gene identification of rhizobia; Table S9: Reference strains for constructing a phylogenetic tree; Table S10: qRT-PCR primers used in this study. Supplementary Data File S1: List of gene IDs enriched in the top 20 GO terms of Module 39; Supplementary Data File S2: List of gene IDs enriched in the top 20 KEGG terms of Module 39; Supplementary Data File S3: List of gene IDs enriched in the top 20 GO terms of Module 8; Supplementary Data File S4: List of gene IDs enriched in the top 20 KEGG terms of Module 8.
