# Supplementary material for: Time-Series Transcriptomics Reveals Temporal Expression Patterns in Soybean (Glycine max) Roots During Symbiosis with Bradyrhizobium ottawaense HK601
Source: Plants (Basel). 2026 Nov 3;15(21):3420. doi: 10.3390/plants15213420 (PMC13765555; doi:10.3390/plants15213420)
Supplement: Supplementary file 1 [file plants-15-03420-s001.zip › Supplementary Tables.pdf]

Table S1. Summary of RNA-seq data quality and alignment statistics

Sample	RawReads (M)	RawBases (G)	CleanReads (M)	CleanBases (G)	ValidBase (%)	Q30 (%)	GC (%)	Total mapped reads	Multiple mapped	Uniquely mapped
0dpi-1	23.88	7.10	23.64	7.03	98.98	98.60	41.51	46540931(98.43%)	1457081(3.08%)	45083850(95.35%)
0dpi-2	24.01	7.12	23.71	7.03	98.77	98.21	39.86	46609671(98.28%)	1127377(2.38%)	45482294(95.91%)
0dpi-3	23.59	7.02	23.38	6.96	99.14	98.42	41.79	45992643(98.34%)	1480150(3.16%)	44512493(95.18%)
2dpi-1	23.57	7.00	23.29	6.91	98.82	98.24	41.34	45751585(98.21%)	1073393(2.30%)	44678192(95.91%)
2dpi-2	23.67	7.02	23.37	6.93	98.73	98.32	40.41	45912843(98.24%)	1295059(2.77%)	44617784(95.47%)
2dpi-3	23.78	7.05	23.46	6.95	98.64	98.46	39.16	46053979(98.17%)	1311427(2.80%)	44742552(95.37%)
7dpi-1	23.84	7.07	23.52	6.97	98.64	98.3	40.57	46237198(98.31%)	1087779(2.31%)	45149419(96.00%)
7dpi-2	24.04	7.13	23.74	7.04	98.74	98.23	39.56	46563361(98.07%)	958237(2.02%)	45605124(96.05%)
7dpi-3	23.63	7.01	23.34	6.93	98.77	98.10	43.93	45870627(98.28%)	1235451(2.65%)	44635176(95.63%)
14dpi-1	23.74	7.06	23.5	6.99	98.99	98.12	42.16	45914801(97.67%)	1261959(2.68%)	44652842(94.99%)
14dpi-2	23.74	7.02	23.36	6.91	98.4	98.31	43.81	45615474(97.62%)	1379638(2.95%)	44235836(94.66%)
14dpi-3	23.81	7.03	23.39	6.9	98.23	98.42	39.81	45809922(97.93%)	1062370(2.27%)	44747552(95.66%)
21dpi-1	24.02	7.13	23.74	7.05	98.83	98.24	42.62	46487556(97.92%)	1717293(3.62%)	44770263(94.31%)
21dpi-2	23.82	7.08	23.57	7	98.93	98.47	41.32	46159044(97.93%)	1507062(3.20%)	44651982(94.73%)

Sample	RawReads (M)	RawBases (G)	CleanReads (M)	CleanBases (G)	ValidBase (%)	Q30 (%)	GC (%)	Total mapped reads	Multiple mapped	Uniquely mapped
21dpi-3	23.85	7.09	23.59	7.01	98.91	98.09	40.88	46073878(97.67%)	1479201(3.14%)	44594677(94.54%)
28dpi-1	23.6	7	23.28	6.9	98.65	98.38	41.71	45581170(97.88%)	1433617(3.08%)	44147553(94.80%)
28dpi-2	24	7.14	23.76	7.07	99	98.07	43.74	46457944(97.78%)	1802798(3.79%)	44655146(93.98%)
28dpi-3	23.55	6.99	23.27	6.91	98.83	98.50	41.68	45583791(97.94%)	1530162(3.29%)	44053629(94.66%)

Table S2. GO enrichment analysis of genes in Module 39

id	Term	Category	ListHits	PopHits	*p*-value	*q*-value	-log₁₀*p*	Enrichment_score	geneID
GO:0006468	protein phosphorylation	BP	201	1986	5.43×10^{-12}	6.16×10^{-9}	11.27	1.62	*SYMRK-BETA, EDR, CRK19, CRK84, CRK74* et al.
GO:0048544	recognition of pollen	BP	26	112	4.36×10^{-9}	2.48×10^{-6}	8.36	3.72	*LOC112998744, LOC100782811, LOC100779455* et al.
GO:0005524	ATP binding	MF	312	3753	5.55×10^{-8}	2.10×10^{-5}	7.26	1.33	*LOC100777348, LOC100777629, LOC100777836* et al.
GO:0009299	mRNA transcription	BP	11	25	1.07×10^{-7}	3.05×10^{-5}	6.97	7.05	*LOC100806497, LOC100803605, LOC100527409* et al.
GO:0016036	cellular response to phosphate starvation	BP	9	18	4.10×10^{-7}	9.31×10^{-5}	6.39	8.01	*PHO1-H10, SPX1, SPX3* et al.
GO:0004672	protein kinase activity	MF	121	1244	8.58×10^{-7}	1.44×10^{-4}	6.07	1.56	*LOC100777348, LOC100781785, LOC100793659* et al.
GO:0003993	acid phosphatase activity	MF	15	55	8.89×10^{-7}	1.44×10^{-4}	6.05	4.37	*LOC100789603, LOC100794124, LOC100803398* et al.
GO:0004497	monooxygenase activity	MF	44	327	1.38×10^{-6}	1.97×10^{-4}	5.86	2.16	*F6H1, I2'H, CYP81E11, CYP98A2* et al.
GO:0009247	glycolipid biosynthetic process	BP	7	12	2.19×10^{-6}	2.76×10^{-4}	5.66	9.35	*LOC100780899, LOC100803792, LOC100794727* et al.
GO:0016705	oxidoreductase activity, acting on paired donors, with incorporation or reduction of molecular oxygen	MF	43	326	3.06×10^{-6}	3.48×10^{-4}	5.51	2.11	*F6H1, LOC100799715, LOC100801007* et al.

id	Term	Category	ListHits	PopHits	*p*-value	*q*-value	$-\log_{10}p$	Enrichment_score	geneID
GO:0080043	quercetin 3-O-glucosyltransferase activity	MF	13	47	3.98×10^{-6}	3.77×10^{-4}	5.40	4.43	*LOC100786860*, *LOC100804361*, *LOC100798148* et al.
GO:0080044	quercetin 7-O-glucosyltransferase activity	MF	13	47	3.98×10^{-6}	3.77×10^{-4}	5.40	4.43	*LOC100786860*, *LOC100804361*, *LOC100798148* et al.
GO:0043565	sequence-specific DNA binding	MF	67	610	5.44×10^{-6}	4.75×10^{-4}	5.26	1.76	*LOC100527466*, *WRKY1*, *WRKY39*, *WRKY19* et al.
GO:0020037	heme binding	MF	68	625	6.39×10^{-6}	5.18×10^{-4}	5.19	1.74	*F6H1*, *LB1*, *LB2*, *LB3*, *LB4*, *LOC100799715* et al.
GO:0005506	iron ion binding	MF	54	471	1.29×10^{-5}	9.80×10^{-4}	4.89	1.84	*F6H1*, *LOC100796655*, *LOC100799715* et al.
GO:0006145	Purine nucleobase catabolic process	BP	5	7	1.78×10^{-5}	1.25×10^{-3}	4.75	11.45	*LOC100796088*, *UR9*, *LOC100809727*, *UR2*, *LOC100795029*
GO:0008194	UDP-glycosyltransferase activity	MF	20	112	1.88×10^{-5}	1.25×10^{-3}	4.73	2.86	*LOC100778639*, *LOC100800400*, *LOC100791935*, *IF7GT3* et al.
GO:0004842	ubiquitin-protein transferase activity	MF	30	214	2.84×10^{-5}	1.79×10^{-3}	4.55	2.25	*LOC100776142*, *LOC100796470*, *LOC100815248* et al.
GO:0000287	magnesium ion binding	MF	26	175	3.47×10^{-5}	2.08×10^{-3}	2.38	2.38	*LOC100777629*, *LOC100787663*, *LOC100803925* et al.
GO:0004674	protein serine/threonine kinase activity	MF	69	676	4.37×10^{-5}	2.32×10^{-3}	1.64	1.64	*LOC100779991*, *LOC100782913*, *LOC100787825* et al.

Notes: The complete gene IDs for each GO term are provided in Supplementary Data File S1.

Table S3. KEGG enrichment analysis of genes in Module 39

id	Term	Classification_level2	ListHits	*p*-value	Enrichment_score	geneID
gmx04016	MAPK signaling pathway - plant	Signal transduction	44	3.28×10^{-7}	2.24	*PR1-6*, *CAT1*,*WRKY39* et al.
gmx00230	Purine metabolism	Nucleotide metabolism	29	1.92×10^{-6}	2.59	*GS52*, *HIUHase*, *LOC100796088*, *LOC100817607* et al.
gmx00270	Cysteine and methionine metabolism	Amino acid metabolism	30	3.64×10^{-5}	2.20	*LOC100800830*, *LOC100806526*, *LOC100818242* et al.
gmx04146	Peroxisome	Transport and catabolism	24	5.67×10^{-5}	2.39	*SODB2*, *LOC121172669*, *LOC100780811* et al.
gmx00630	Glyoxylate and dicarboxylate metabolism	Carbohydrate metabolism	21	9.98×10^{-5}	2.47	*GS1-GAMMA*, *GS1GAMMA2*, *LOC100305381* et al.
gmx00740	Riboflavin metabolism	Metabolism of cofactors and vitamins	10	1.32×10^{-4}	3.98	*LOC100778763*, *LOC100789603*, *LOC100795684* et al.
gmx00943	Isoflavonoid biosynthesis	Biosynthesis of other secondary metabolites	11	5.47×10^{-4}	3.16	*F6H1*, *LOC100781863*, *LOC100793687* et al.
gmx00566	Sulfoquinovose metabolism	Carbohydrate metabolism	6	1.24×10^{-3}	4.66	*LOC100780899*, *LOC100803792*, *LOC100788781* et al.
gmx00920	Sulfur metabolism	Energy metabolism	11	1.38×10^{-3}	2.85	*LOC100813286*, *LOC100818242*, *LOC606297* et al.
gmx00250	Alanine, aspartate and glutamate metabolism	Amino acid metabolism	14	2.16×10^{-3}	2.36	*GS1-GAMMA*, *GS1GAMMA2*, *LOC100796941* et al.
gmx02010	ABC transporters	Membrane transport	17	6.37×10^{-3}	1.94	*LOC100777836*, *LOC100794527*, *LOC100795627* et al.
gmx00030	Pentose phosphate pathway	Carbohydrate metabolism	14	7.90×10^{-3}	2.05	*LOC100794730*, *LOC100786514*, *LOC100798766* et al.

id	Term	Classification_level2	ListHits	*p*-value	Enrichment_score	geneID
gmx00950	Isoquinoline alkaloid biosynthesis	Biosynthesis of other secondary metabolites	8	8.39×10^{-3}	2.70	*LOC100785410*, *LOC100800830*, *LOC100801369* et al.
gmx00010	Glycolysis / Gluconeogenesis	Carbohydrate metabolism	27	8.53×10^{-3}	1.62	*LOC100787663*, *LOC100788951*, *LOC100803925* et al.
gmx00710	Carbon fixation by Calvin cycle	Energy metabolism	16	9.24×10^{-3}	1.91	*PEPC7*, *LOC100804853*, *LOC100799358* et al.
gmx04626	Plant-pathogen interaction	Environmental adaptation	43	1.05×10^{-2}	1.43	*LOC100809298*, *PR1-6*, *LOC100800248* et al.
gmx00561	Glycerolipid metabolism	Lipid metabolism	18	1.08×10^{-2}	1.80	*DGD1*, *LOC100780899*, *LOC100788951* et al.
gmx00130	Ubiquinone and other terpenoid-quinone biosynthesis	Metabolism of cofactors and vitamins	13	1.17×10^{-2}	2.02	*LOC100790396*, *LOC100800830*, *LOC100788328* et al.
gmx00220	Arginine biosynthesis	Amino acid metabolism	9	1.55×10^{-2}	2.29	*GS1-GAMMA*, *GS1GAMMA2*, *LOC100777694* et al.
gmx00592	alpha-Linolenic acid metabolism	Lipid metabolism	12	1.72×10^{-2}	1.98	*LOC100800036*, *LOC100780811*, *LOC100790627* et al.

Notes: The complete gene IDs for each KEGG term are provided in Supplementary Data File S2.

Table S4. GO enrichment analysis of genes in Module 8

id	Term	Category	ListHits	PopHits	*p*-value	*q*-value	-log$_{10}p$	Enrichment_score	geneID
GO:0003735	structural constituent of ribosome	MF	425	610	2.25×10^{-157}	4.31×10^{-154}	156.65	3.47	*LOC100305645; LOC100815850; LOC100306058* et al.
GO:0005840	ribosome	CC	369	528	3.73×10^{-137}	3.58×10^{-134}	136.43	3.48	*LOC100305645; LOC100815850; LOC100306058* et al.
GO:0022625	cytosolic large ribosomal subunit	CC	205	230	1.02×10^{-113}	6.55×10^{-111}	112.99	4.43	*LOC100306058; LOC100499931; LOC100802599* et al.
GO:0006412	translation	BP	325	499	2.27×10^{-107}	1.09×10^{-104}	106.64	3.24	*LOC100305645; LOC100815850; LOC100499931* et al.
GO:0022627	cytosolic small ribosomal subunit	CC	117	138	4.66×10^{-60}	1.79×10^{-57}	59.33	4.22	*LOC100305645; LOC100815850; LOC121173836* et al.
GO:0008017	microtubule binding	MF	158	292	5.06×10^{-38}	1.62×10^{-35}	37.30	2.69	*LOC100776414; LOC100788335; LOC100784439* et al.
GO:0002181	cytoplasmic translation	MF	68	78	4.48×10^{-37}	1.23×10^{-34}	36.35	4.34	*LOC100306058; LOC100499952; LOC100305556* et al.
GO:0007018	microtubule-based movement	BP	93	139	6.09×10^{-33}	1.46×10^{-30}	32.22	3.33	*LOC100788335; LOC100784439; LOC100784714* et al.
GO:0003777	microtubule motor activity	BP	82	120	3.24×10^{-30}	6.90×10^{-28}	29.49	3.40	*LOC100788335; LOC100784439; LOC100775206* et al.
GO:0000307	cyclin-dependent protein kinase holoenzyme complex	CC	66	126	8.46×10^{-16}	1.62×10^{-13}	15.07	2.61	*LOC100805119; LOC547740; LOC100812029* et al.
GO:0000028	ribosomal small subunit assembly	BP	31	41	3.08×10^{-14}	5.37×10^{-12}	13.51	3.76	*LOC100815850; LOC100499781; LOC100809054* et al.
GO:0044772	mitotic cell cycle phase	BP	43	72	2.07×10^{-13}	3.26×10^{-11}	12.68	2.97	*LOC547740; LOC100812029;*

id	Term	Category	ListHits	PopHits	*p*-value	*q*-value	$-\log_{10}p$	Enrichment_score	geneID
	transition								*LOC100776207* et al.
GO:0000079	regulation of cyclin-dependent protein serine/threonine kinase activity	BP	50	91	2.21×10^{-13}	3.26×10^{-11}	12.66	2.73	*LOC547740; LOC100812029; LOC100776207* et al.
GO:0000027	ribosomal large subunit assembly	BP	30	42	9.90×10^{-13}	1.36×10^{-10}	12.00	3.55	*LOC100817746; LOC100527754; LOC100500064* et al.
GO:0016538	cyclin-dependent protein serine/threonine kinase regulator activity	MF	44	79	3.19×10^{-12}	4.08×10^{-10}	11.50	2.77	*LOC547740; LOC100812029; LOC100776207* et al.
GO:0010088	phloem development	BP	18	19	4.33×10^{-12}	5.19×10^{-10}	11.36	4.71	*SEO-F1; SEO-F2; SEOU* et al.
GO:0006270	DNA replication initiation	BP	22	27	1.27×10^{-11}	1.43×10^{-9}	10.90	4.05	*LOC100814940; LOC100779483; LOC100791200* et al.
GO:0042273	ribosomal large subunit biogenesis	BP	29	43	2.29×10^{-11}	2.44×10^{-9}	10.64	3.36	*LOC100817659; LOC100499746; LOC100808592* et al.
GO:0003723	RNA binding	MF	312	1135	7.26×10^{-10}	7.33×10^{-8}	9.14	1.37	*LOC100807513; LOC100781647; LOC100806355* et al.
GO:0005874	microtubule	CC	47	100	1.32×10^{-9}	1.27×10^{-7}	8.88	2.34	*LOC100776414; LOC100779567; LOC100779826* et al.

Notes: The complete gene IDs for each GO term are provided in Supplementary Data File S3.

Table S5. KEGG enrichment analysis of genes in Module 8

id	Term	Classification_level2	ListHits	*p*-value	Enrichment_score	geneID
gmx03010	Ribosome	Translation	424	3.89×10^{-141}	3.02	*LOC100305645; LOC100815850; LOC100306058* et al.
gmx04814	Motor proteins	Cell motility	97	2.83×10^{-24}	2.61	*LOC100781142; LOC100817933; LOC100784714* et al.
gmx03030	DNA replication	Replication and repair	40	3.50×10^{-10}	2.53	*LOC100777091; LOC100798779; LOC548079* et al.
gmx04517	IgSF CAM signaling	Signaling molecules and interaction	47	1.89×10^{-8}	2.13	*LOC100794287; LOC100799091; LOC100805460* et al.
gmx05100	Bacterial invasion of epithelial cells	Infectious disease: bacterial	26	2.58×10^{-6}	2.36	*LOC100794287; LOC100799091; LOC100805460* et al.
gmx00240	Pyrimidine metabolism	Nucleotide metabolism	46	9.96×10^{-5}	1.67	*LOC100500294; LOC547948; LOC100798205* et al.
gmx04145	Phagosome	Transport and catabolism	56	3.23×10^{-4}	1.53	*LOC100794287; LOC100799091; LOC100805460* et al.
gmx00510	N-Glycan biosynthesis	Glycan biosynthesis and metabolism	33	5.49×10^{-4}	1.71	*LOC100816018; LOC100814831; LOC100805094* et al.
gmx04820	Cytoskeleton in muscle cells	Cell motility	16	1.67×10^{-3}	2.05	*LOC100786139; LOC100527547; LOC100305720* et al.
gmx00513	Various types of N-glycan biosynthesis	Glycan biosynthesis and metabolism	27	2.32×10^{-3}	1.68	*LOC100816018; LOC100786953; LOC100814831* et al.
gmx03050	Proteasome	Folding, sorting and degradation	36	6.14×10^{-3}	1.48	*LOC100785863; LOC100804455; LOC100802514* et al.
gmx03008	Ribosome biogenesis in	Translation	47	8.21×10^{-3}	1.38	*LOC100775298; LOC100806355;*

id	Term	Classification_level2	ListHits	*p*-value	Enrichment_score	geneID
	eukaryotes					*LOC100815506* et al.
gmx03430	Mismatch repair	Replication and repair	19	9.18×10^{-3}	1.69	*LOC100777091; LOC548079; LOC100817651* et al.
gmx03420	Nucleotide excision repair	Replication and repair	41	1.11×10^{-2}	1.40	*LOC100777091; LOC548079; LOC100817651* et al.
gmx03440	Homologous recombination	Replication and repair	26	1.14×10^{-2}	1.53	*LOC100777091; LOC100804958; LOC100793247* et al.
gmx03410	Base excision repair	Replication and repair	26	1.95×10^{-2}	1.47	*LOC100810677; LOC100793449; LOC100813637* et al.
gmx00061	Fatty acid biosynthesis	Lipid metabolism	27	2.85×10^{-2}	1.42	*LOC100807065; LOC100777962; LOC548028* et al.
gmx04620	Toll-like receptor signaling pathway	Immune system	11	2.94×10^{-2}	1.78	*LOC100794287; LOC100799091; LOC100805460* et al.
gmx04144	Endocytosis	Transport and catabolism	79	5.01×10^{-2}	1.18	*LOC100791688; LOC100789188; LOC100809091* et al.
gmx03450	Non-homologous end-joining	Replication and repair	5	5.62×10^{-2}	2.18	*LOC100775475; LOC100792878; LOC100797038* et al.

Notes: The complete gene IDs for each KEGG term are provided in Supplementary Data File S4

Table S6. Expression levels of genes enriched in the isoflavonoid biosynthesis pathway

NO.	Gene id	0 dpi	2 dpi	7 dpi	14 dpi	21 dpi	28 dpi
1	*F6H1*	0	1.61	5.09	7.10	9.20	9.24
2	*LOC100781863*	0	0.73	2.13	2.53	3.70	3.01
3	*LOC100793687*	0	1.11	3.38	3.95	4.69	3.97
4	*I2′H*	0	1.19	4.39	4.88	8.31	5.96
5	*CYP81E11*	0	0.54	1.75	2.65	2.62	3.19
6	*IF7GT*	0	1.93	1.86	3.47	4.29	4.17
7	*LOC100797371*	0	0.19	2.41	2.89	5.94	4.15
8	*IF7MaT*	0	-0.11	0.44	0.55	0.99	1.03
9	*HIDH*	0	-0.14	0.81	1.33	1.48	1.01
10	*LOC100819796*	0	2.51	5.65	6.70	6.87	8.29
11	*LOC100804509*	0	1.34	3.51	4.55	5.53	4.02

Note: Data represent log_2(fold change) relative to the 0 dpi control.

Table S7. Expression levels of genes enriched in the MAPK signaling pathway - plant

NO.	Gene id	0 dpi	2 dpi	7 dpi	14 dpi	21 dpi	28 dpi
1	*LOC100777324*	0	2.4	2.13	3.7	9.31	10.81
2	*LOC100779954*	0	1.35	0	5.42	9.81	10.67
3	*LOC100787825*	0	0	0	11.82	15.34	15.29
4	*LOC100798037*	0	0.39	0.76	1.48	1.86	2.46
5	*PR1-6*	0	4.14	6.50	6.89	10.04	10.95
6	*LOC100800248*	0	0.96	1.10	1.37	1.36	1.74
7	*WRKY39*	0	0.48	0.92	1.36	1.80	1.25
8	*WRKY19*	0	1.52	1.81	2.27	2.65	2.69
9	*WRKY62*	0	1.23	1.42	2.46	2.91	3.00
10	*LOC100807068*	0	-0.19	2.24	2.61	2.26	2.59
11	*WRKY49*	0	0.22	0.89	1.12	1.65	1.2
12	*PR1-7*	0	3.36	6.44	7.27	8.45	8.43
13	*CAT4*	0	-0.24	-0.53	1.30	2.64	2.47
14	*LOC106794811*	0	0	0	5.76	7.96	6.97
15	*LOC100776837*	0	0.77	1.18	1.57	1.85	1.80
16	*LOC100807981*	0	0.34	1.08	1.59	1.27	1.62
17	*LOC100792833*	0	0.24	0.79	1.01	1.87	1.42
18	*LOC100789164*	0	1.13	2.69	4.55	3.04	5.44
19	*LOC100793600*	0	-0.16	0.43	0.81	0.93	1.54
20	*LOC100808416*	0	0.07	0.86	1.73	1.07	2.59
21	*LOC100817431*	0	0.78	1.48	1.59	1.56	2.68
22	*LOC100787949*	0	0.10	1.19	1.68	1.22	1.76
23	*LOC100776289*	0	0.07	0.58	0.95	1.15	1.36
24	*LOC100813603*	0	0.60	1.35	1.11	1.61	1.79
25	*LOC100527909*	0	0.28	0.22	0.63	0.58	0.68
26	*CAT1*	0	0.24	0.48	0.89	1.03	1.21
27	*LOC100804100*	0	0.24	0.64	1.14	0.97	1.59
28	*LOC100799682*	0	0.31	0.93	1.34	0.92	1.54
29	*LOC100819999*	0	0.13	0.42	0.60	0.64	0.79
30	*LOC121174020*	0	0.43	0.58	1.15	0.86	1.08
31	*LOC100788662*	0	0.64	1.70	2.12	1.54	2.56
32	*LOC100788199*	0	0.13	0.47	0.93	0.55	1.35
33	*LOC100798803*	0	0.30	0.27	0.64	1.13	0.84
34	*PRI1-9*	0	0	0	3.34	4.79	6.58
35	*LOC100790168*	0	0.01	0.47	0.58	0.62	1.07
36	*LOC100790854*	0	0.09	0.61	1.01	0.75	1.10
37	*LOC100805418*	0	0.42	0.55	0.92	0.79	1.00
38	*GRR1*	0	0.40	0.44	0.81	0.73	0.91
39	*LOC100807275*	0	0.46	0.59	1.84	2.47	2.10
40	*LOC100780576*	0	0.65	2.66	3.32	5.41	4.15
41	*LOC100814917*	0	1.55	1.26	1.46	2.86	2.99
42	*LOC100791624*	0	0.96	2.76	3.37	3.91	4.59

43	*PR1-1*	0	0	0	2.45	2.94	5.88
44	*LOC100820247*	0	0.06	0.11	0.18	0.14	0.30

Note: Data represent log_2(fold change) relative to the 0 dpi control.

Table S8. Primers for gene identification of rhizobia

primer name	Primer sequence (5'–3')
16s 27F	AGAGTTTGATCCTGGCTCAG
16s 1492R	AAGGAGGTGATCCAGCC
atpD 225F	GCTSGGCCGCATCMTSAACGT
atpD 782R	GCCGACACTTCMGAACNNGCCTG
gln II 12F	GCCGACACTTCMGAACNNGCCTG
gln II 689R	TGCATGCCSGAGCCGTTCCA
recA 41F	TTCGGCAAGGGMTCGRTSATG
recA 640R	ACATSACRCCGATCTTCATGC

Table S9. Reference strains for constructing a phylogenetic tree

Number	Strains name	GenBank			
		***16S* rDNA**	***atpD***	***gln II***	***recA***
1	HK601	PX496391	PZ567848	PZ567849	PZ567850
2	*Sinorhizobium meliloti* 1021^T	/	AL591688	AL591985	AL591688
3	*Bradyrhizobium paxllaeri LMTR21T*	AY923031	KF896186	KF896169	JX943617
4	*Bradyrhizobium cytisi CTAW11T*	EU561065	GU001613	GU001594	GU001575
5	*Bradyrhizobium huanghuaihaiense CCBAU 23303T*	HQ231463	HQ231682	HQ231639	HQ231595
6	*Bradyrhizobium japonicum USDA 6T*	AB231927	AJ294388	AF169582	AM168341
7	*Bradyrhizobium ottawaense OO99T*	JN186270	HQ455212	HQ587750	HQ587287
8	*Bradyrhizobium oligotrophicum LMG 10732T*	JQ619230	AP012603	AP012603	AP012603
9	*Bradyrhizobium icense LMTR13T*	KF896156	KF896192	KF896175	JX943615
10	*Bradyrhizobium lupini USDA 3051T*	KM114861	KU738808	KM114862	KM114866
11	*Bradyrhizobium elkanii USDA 76T*	U35000	AY386758	AY599117	AM168342
12	*Bradyrhizobium denitrificans LMG 8443T*	X66025	FM253153	HM047121	FM253196

Table S10. qRT-PCR primers used in this study

primer name	Primer sequence (5'–3')
LB1-F	GTGATTGATGCCGCACTTGG
LB1-R	CTGCTCAACTCGTCACTCCA
LB2-F	GGCTGATGCTGCACTTGTTT
LB2-R	AGGCTACTTCCCAAGCACTG
N44-F	GCTGAGGCGTATGAGTCTCC
N44-R	AAGGCAAAGAGCCAACCCAT
N26B-F	AGGGTTGGCTCTTTGCCTTT
N26B-R	TGGGAGGTATGGGTGGTGAT
N20-F	GAGAAGTGCTTGGCAGACCA
N21-R	TGCACTGACATGGGAACAGG
N20-F	ATGGGAAGCAAACCCTAGCC
N20-R	CCAACCCATCATTGGCTCCT
