# Supplementary material for: Time-Series Transcriptomics Reveals Temporal Expression Patterns in Soybean (Glycine max) Roots During Symbiosis with Bradyrhizobium ottawaense HK601
Source: Plants (Basel). 2026 Nov 3;15(21):3420. doi: 10.3390/plants15213420 (PMC13765555; doi:10.3390/plants15213420)
Supplement: Supplementary file 1 [file plants-15-03420-s001.zip › Supplementary Figures.pdf]

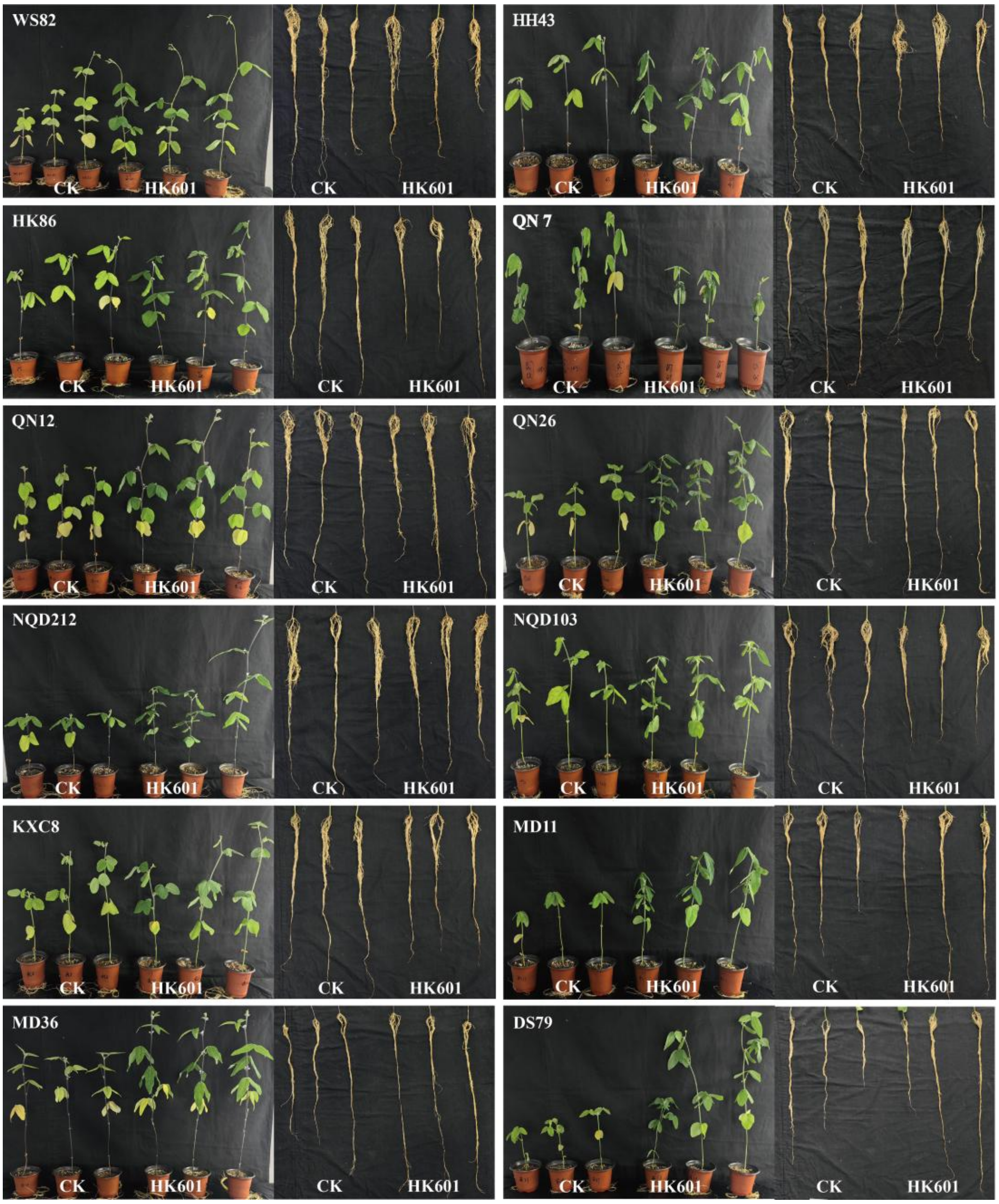

Figure S1. Broad-spectrum nodulation phenotype of *B. ottawaense* HK601 in pot experiments

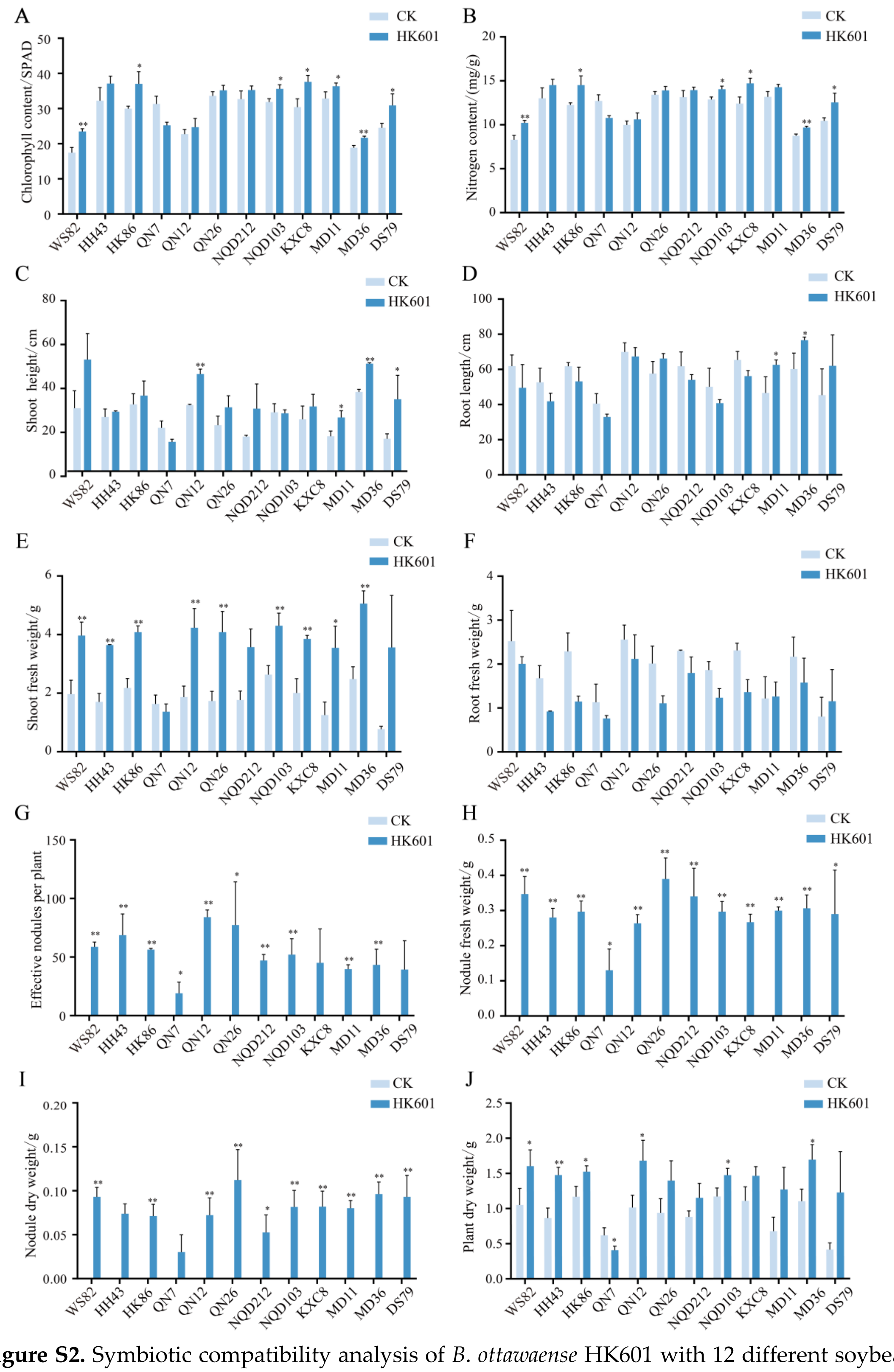

Figure S2. Symbiotic compatibility analysis of *B. ottawaense* HK601 with 12 different soybean cultivars. (A) Leaf chlorophyll content (SPAD value). (B) Leaf nitrogen content. (C) Shoot height. (D) Root length. (E) Shoot fresh weight. (F) Root fresh weight (excluding nodules). (G) Number of effective nodules per plant. (H) Fresh weight of effective nodules. (I) Dry weight of effective nodules. (J) Plant dry weight. Data were presented as means ± SD ($n = 3$, one plant per pot). * and ** indicate significant differences at $p < 0.05$ and $p < 0.01$ levels, respectively.

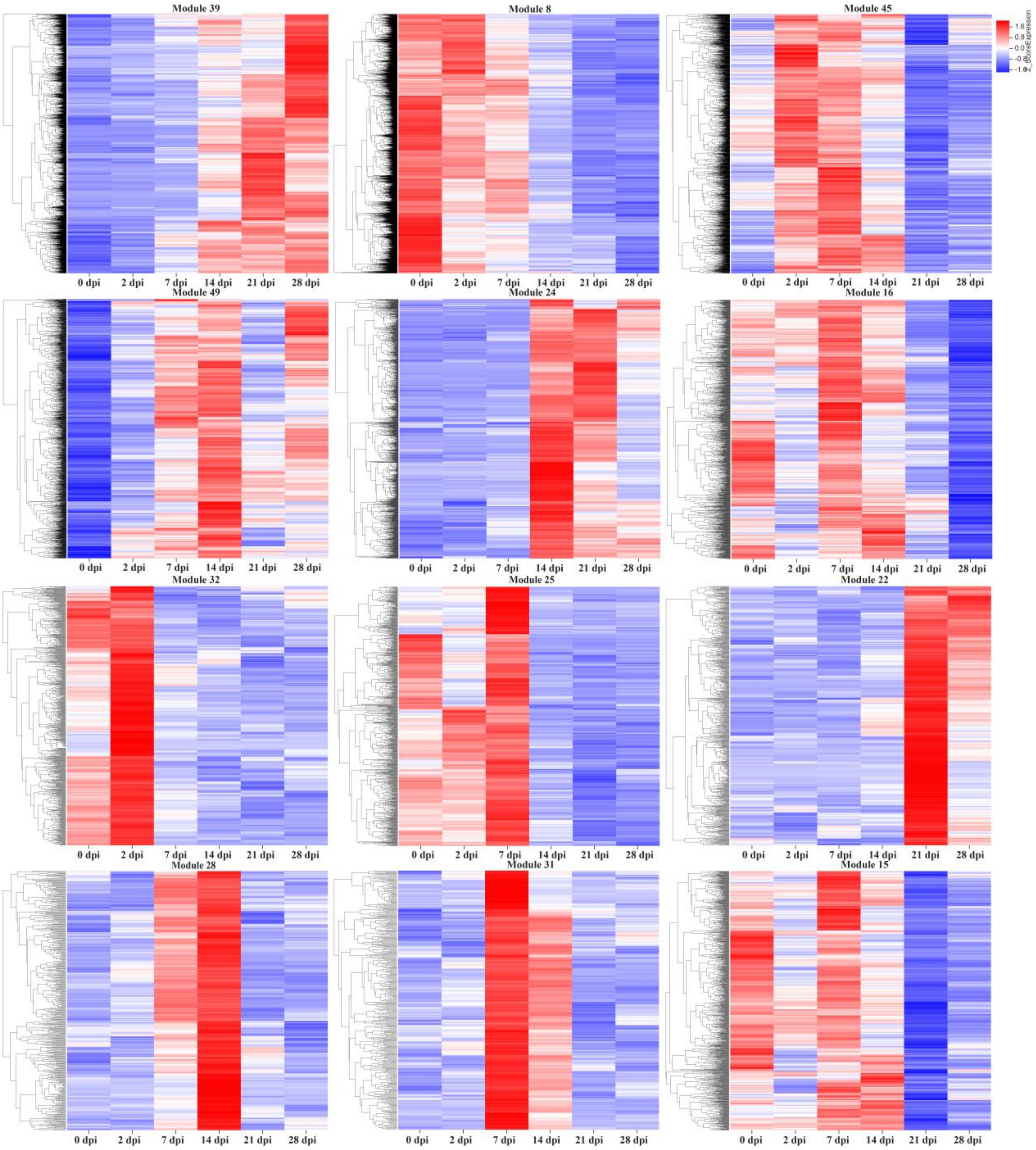

Figure S3. Trend clustering heatmap of differentially expressed genes

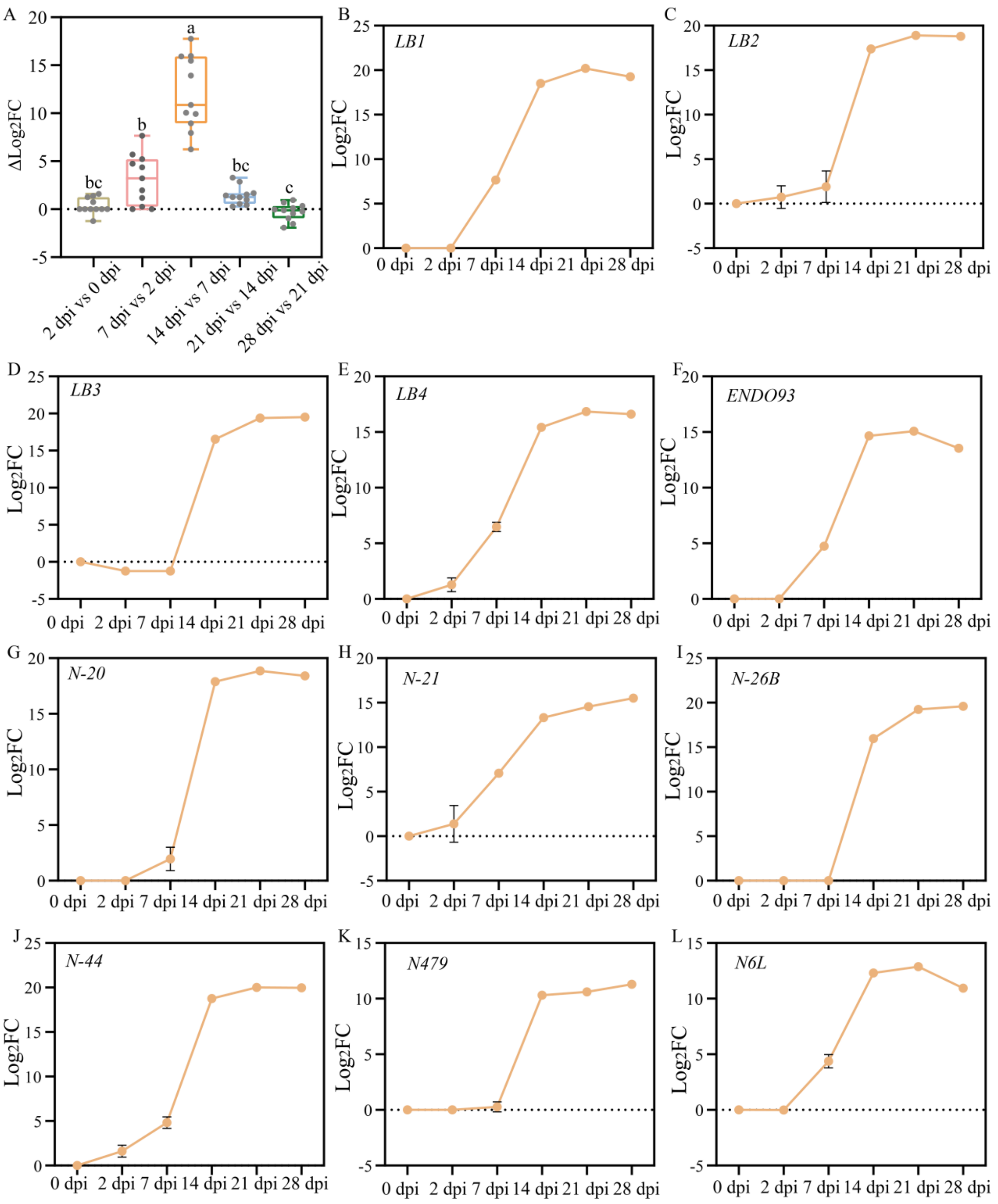

Figure S4. Temporal expression dynamics and statistical validation of symbiotic marker genes across all time points. (A) Boxplot illustrating the distribution of adjacent expression changes (ΔLog_2FC) for the 11 symbiotic marker genes. To control for inherent baseline differences among genes, individual genes were treated as paired statistical units ($n = 11$) to evaluate intra-gene expression shifts across intervals. The box represents the 25th – 75th percentiles (IQR), the horizontal line indicates the median, and whiskers extend to the minimum and maximum values (Tukey method). Each dot represents an individual marker gene. Different lowercase letters (a, b, bc, c) denote significant differences between adjacent intervals (repeated-measures one-way ANOVA followed by Tukey's multiple comparisons test, $p < 0.05$). (B-L) Transcriptional trajectories (Log_2FC, calculated relative to the 0 dpi baseline) of 11 individual symbiotic marker genes (*LB1*, *LB2*, *LB3*, *LB4*, *ENOD93*, *N-20*, *N-21*,

N-26B, *N-44*, *N479*, and *N6L*) from 0 to 28 dpi. Data are presented as mean $\pm$ SD derived from three independent biological replicates.
